# Is there a relationship between geographic distance and uptake of HIV testing services? A representative population-based study of Chinese adults in Guangzhou, China

**DOI:** 10.1371/journal.pone.0180801

**Published:** 2017-07-20

**Authors:** Wen Chen, Fangjing Zhou, Brian J. Hall, Joseph D. Tucker, Carl Latkin, Andre M. N. Renzaho, Li Ling

**Affiliations:** 1 Faculty of Medical Statistics and Epidemiology, School of Public Health, Sun Yat-sen University, Guangzhou, Guangdong, China; 2 Center for Migrant Health Policy, Sun Yat-sen University, Guangzhou, Guangdong, China; 3 School of Social Science and Psychology, Western Sydney University, Penrith, New South Wales, Australia; 4 Department of Prevention and Treatment, Center for Tuberculosis Control of Guangdong Province, Guangzhou, Guangdong, China; 5 Global and Community Mental Health Research Group, Faculty of Social Sciences, Department of Psychology, University of Macau, Macau, China; 6 Department of Health Behavior and Society, Johns Hopkins Bloomberg School of Public Health, Baltimore, Maryland, United States of America; 7 UNC-Project China, Guangzhou, Guangdong, China; 8 UNC Global Health and Infectious Diseases, University of North Carolina at Chapel Hill, Chapel Hill, North Carolina, United States of America; 9 Department of Epidemiology, Johns Hopkins Bloomberg School of Public Health, Baltimore, Maryland, United States of America; Public Health Agency of Canada, CANADA

## Abstract

Achieving high coverage of HIV testing services is critical in many health systems, especially where HIV testing services remain centralized and inconvenient for many. As a result, planning the optimal spatial distribution of HIV testing sites is increasingly important. We aimed to assess the relationship between geographic distance and uptake of HIV testing services among the general population in Guangzhou, China. Utilizing spatial epidemiological methods and stratified household random sampling, we studied 666 adults aged 18–59. Computer-assisted interviews assessed self-reported HIV testing history. Spatial scan statistic assessed the clustering of participants who have ever been tested for HIV, and two-level logistic regression models assessed the association between uptake of HIV testing and the mean driving distance from the participant’s residence to all HIV testing sites in the research sites. The percentage of participants who have ever been tested for HIV was 25.2% (168/666, 95%CI: 21.9%, 28.5%), and the majority (82.7%) of participants tested for HIV in Centres for Disease Control and Prevention, public hospitals or STIs clinics. None reported using self-testing. Spatial clustering analyses found a hotspot included 48 participants who have ever been tested for HIV and 25.8 expected cases (Rate Ratio = 1.86, *P* = 0.002). Adjusted two-level logistic regression found an inverse relationship between geographic distance (kilometers) and ever being tested for HIV (aOR = 0.90, 95%CI: 0.84, 0.96). Married or cohabiting participants (aOR = 2.14, 95%CI: 1.09, 4.20) and those with greater social support (aOR = 1.04, 95%CI: 1.01, 1.07) were more likely to be tested for HIV. Our findings underscore the importance of considering the geographical distribution of HIV testing sites to increase testing. In addition, expanding HIV testing coverage by introducing non-facility based HIV testing services and self-testing might be useful to achieve the goal that 90% of people living with HIV knowing their HIV status by the year 2020.

## Introduction

Globally, the general population and at-risk populations comprised approximately 64% and 36% of the 1.9 million new adult HIV infections in 2014 respectively [1]. In many countries, a high prevalence of risky sexual behaviours among the general population has been reported. For example, in the United States, China, and South Africa, the prevalence of multiple sexual partnerships has been estimated at around 15% [2–4]. In India, the prevalence of inconsistent condom use with non-regular sex partners in the last 12 months was estimated at 58% [5]. In addition, emerging evidence suggests that increased global immigration in recent years may have facilitated the spread of the human immunodeficiency virus (HIV) infection [6–8]. As a critical linkage pathway to HIV care and antiretroviral treatment, HIV testing services (HTS) have been recognized as highly cost effective in reducing transmission among at-risk populations [9,10] and the general population [11]. Increasing HTS access decreases morbidity and mortality among people living with HIV [12,13].

It is estimated that, globally, approximately 40% of people living with HIV remain unaware of their status [1]. Given the significant gap between the current estimate and the goal to diagnose 90% of all people living with HIV by 2020 [14], there was an urgent need for widespread HTS, as a fundamental approach to achieve the first goal of the UNAIDS 90-90-90 target [14]. However, HTS remain centralized and inconvenient in many countries, therefore, limiting access to and coverage of HTS [15]. Consequently, the World Health Organization (WHO) has recommended the decentralization of the services whereby HTS are provided in peripheral health facilities and community-based venues, or HIV self-testing with referrals for further HTS are provided [1,15]. However, further research is needed to understand impediments to HIV testing.

China provides a unique opportunity to examine decentralization of HTS because of its largely facility-based HIV testing systems. In 2003, the Chinese government announced the “Four Free and One Care” policy [16]. Under this policy, free voluntary counseling and testing and antiretroviral treatment are provided in a limited number of government designated testing facilities, such as Centres for Disease Control and Prevention (CDC), authorized public hospitals, and specialized public sexually transmitted infections (STIs)/dermatology clinics. In order to expand HTS in China, provider-initiated HIV testing and counseling have been launched and focused on the integration of HIV testing with other routine medical care [17]. However, the services have been predominantly facility-based. Given the urgent need for convenient and discreet HIV testing approaches, HIV self-testing has firstly been implemented among key populations [15,18], and then piloted in the general population [19]. Despite the increase in HTS coverage during the last decade, especially the expansion of centralized facility-based HIV testing program [20,21], the uptake of HIV testing remains suboptimal in China. Among men who have sex with men (MSM) and female migrants working in entertainment venues, HIV testing rates have been under 30% [22,23], and the median of annual HIV testing rates among the general population at China comprehensive AIDS response program sites was 5.5% in 2011 [24].

Geographic distance, as an important indicator of physical accessibility to existing HTS, may affect an individual's decision to obtain an HIV testing. Acton suggested that distance functions as a cost in determining the demand for medical care even when the service is free [25]. Previous studies also found that distance is a significant factor in patients' healthcare and hospital utilization [26,27]. Identifying the association between geographic distance and the uptake of HIV testing could help to improve planning for new testing site location, and inform future decentralization of HTS.

Therefore, the current study sought to assess the role of geographic distance on HIV testing utilization among community-dwelling adults based on a population-representative survey conducted in Guangzhou, China. We hypothesized that geographic distance is a significant barrier to the uptake of HIV testing. We also examined possible correlates that may explain testing utilization and possible confounders of the association between distance and the uptake of testing. We included engaging in risky sexual behaviour, social support, and participant characteristics as they are all associated with HIV testing utilization in previous studies [28–32].

## Materials and methods

### Setting and study population

Data are from the Guangzhou Health Study, a population-representative epidemiological investigation into the mental, physical, and sexual health of migrants and residents living in two districts of Guangzhou, China, the capital city of Guangdong Province [33,34]. Guangzhou is one of the most prosperous regions and one of the largest cities of internal and international migrants in China [35], and has the highest burden of HIV in Guangdong Province [36], and by the end of 2015, 41,413 people were reported to be living with HIV/AIDS [37].

Stratified cluster sampling was used to select adults, aged between 18 and 59 years old, living in Yuexiu and Tianhe districts in Guangzhou between April and November 2014. Yuexiu and Tianhe are two representative districts in Guangzhou with a similar population density based on comparable residential areas [33], and they are the old and new centre of the city respectively. According to the existing evidence [33], the prevalence of risky sexual behaviours in these two districts varies between 5.1% (used substance before sex) and 50% (rarely or never used a condom when having sex with partners). Populations with high levels of risky sexual behaviours were mainly concentrated in Tianhe district.

Seven hundred geographic coordinates within each district were randomly selected using Geographic Information System (GIS), and overlaid on the most recent Google Earth images to locate the nearest residential building. In order to minimize spatial dependency among observations, only one household, defined as the people resident in the same dwelling, was randomly selected within each chosen building, and no more than one eligible individual was selected within each participating household. The eligibility criteria included: 1) aged 18–59 years old; and 2) having the earliest birthday date within the calendar year. If the eligible interviewee refused to participate, or there was no eligible interviewee in that household, we moved to the next household for no more than thrice within the same building. Households were marked as full/partial completions, refusals, or non-responses wherein non-responses referred to households with nobody responding thrice.

The survey was administered in Mandarin and Cantonese using mobile tablet devices. Computer-assisted self-interview (CASI) was used to collect data on sensitive items, such as sexual behaviours and HIV testing history. To maximally protect the privacy of respondents, other persons, excluding trained interviewers, in that household were asked to leave the room, and their data was uploaded to a secure server cloud immediately by respondents themselves when the interviews were finished. A computer-assisted personal interview (CAPI) is also offered to participants who asked interviewers for help. Additional details of this study protocol have been described previously [33].

### Measurement

The study’s dependent variable was the self-reported lifetime HIV testing (ever-tested or never-tested).

The independent variable was the mean driving distance from the participant’s residence to all HIV testing sites in the study districts. The driving distances in kilometres between the participant’s residence and each testing site were calculated using Google Earth. HIV testing facilities included CDC/ STIs clinic, including CDC, authorized public hospitals or specialized STIs/dermatology clinics; private practices; blood donation centres; self-testing kits purchased online or at a pharmacy; as well as other testing sites. The location of all HIV testing sites in Yuexiu district (*n* = 5) and Tianhe district (*n* = 5) was provided by the public health authority responsible for STI/HIV control. In the current study, none of the participants used self-testing, therefore, the distance was not calculated for this subgroup.

Confounding variables included individual- and subdistrict-level variables. The individual-level confounders included risky sexual behaviours, alcohol use disorder, social support, and demographic characteristics. Risky sexual behaviours in the past 12 months included at least one of the following behaviours: multiple sexual partnerships (had two or more partners), condomless sex (rarely or never used a condom when having sex with at least one of the concurrent partners), anal intercourse, and substance use before sex.

Alcohol use disorder was measured by the Alcohol Use Disorders Identification Test (AUDIT) [38]. The AUDIT tabulates scores from 0 to 40, with low-risk drinking (score 0–7), hazardous level (8–15), harmful level (16–19), or high-risk drinking or alcohol dependence (≥20). It has a high internal consistency reliability index among the general population in China (α = 0.83) [39] and in this study (α = 0.81).

Social support was measured by the Social Support Rating Scale. The scale was developed by Xiao for measuring perceived and received social support among Chinese populations [40,41]. A higher total score indicates greater social support. In the current study, its internal consistency reliability was 0.74.

Socio-demographic characteristics included self-reported age, sex, education level, marital and employment status, monthly personal income, migration status (migrants have no household registration status in Guangzhou), duration of living in Guangzhou, and current sexual status (sexually active in the past 12 months).

The subdistrict-level confounders included characteristics of each study subdistrict (*Jie Dao* in Chinese). These include the total population; the proportion of men, 15–64 years old, college graduates, unmarried, employment, and migrants; the number of health facilities per 1000 population; and the number of entertainment venues per 1000 population. The data was collected and provided by the School of Geography and Planning of Sun Yat-sen University.

### Data analysis

Descriptive statistics including the mean, standard deviation (SD), median, interquartile range, frequency, and percentage were used to summarize the characteristics of the study participants and subdistricts. Differences between participants’ HIV testing status by study variables was assessed by the *t* test or rank sum test for continuous variables or the chi-square test for categorical variables.

The spatial scan statistics (SaTScan) 9.4 software [42] was used to detect spatial clusters of participants who have ever been tested for HIV. SaTScan scanned a circular window across the map with varying radius from zero to an upper limit of 50% of the entire study area and compared the number of observed and expected cases within the window at each location. For each potential cluster, a likelihood ratio test was used to determine if the number of observed participants who have ever been tested for HIV within the cluster was significantly higher than expected. The numbers of expected cases were calculated on the basis of the null hypothesis that people have been tested for HIV was randomly distributed in space and followed a Bernoulli distribution. Rate Ratio was obtained by dividing the number of observed cases by the number of expected cases within each cluster. Hotspots, which were defined as clusters having more observed participants than expected, had rate ratio significantly greater than one (*P*<0.05). On the contrary, coldspots had rate ratio significantly smaller than one. TheQGIS 2.18.10 [43] software was used to translate the significant spatial clusters generated by SaTScan into maps. In addition, we used QGIS 2.18.10 software with the Spatial Analyst Extension to create a continuous map surface. The Inverse Distance Weighting algorithm was used for spatial interpolation.

In this study, the structure of our data was hierarchical, that is, participants (level-1) were nested within subdistricts (level-2). Therefore, two-level logistic regression models were used to assess the associations between ever being tested for HIV and study variables. Firstly, bivariate two-level logistic regression models were performed by including significant(*P*<0.05) confounding variables found in bivariate analysis to estimate unadjusted odds ratios (uOR), and 95% confidence intervals (95%CI). A multivariate two-level logistic regression model was then built to calculate adjusted odds ratios (aOR) and 95% CI. Subdistricts were defined as cluster for the random intercept model in this analysis. Intra-class correlation coefficient (ICC) for ever being tested for HIV was calculated to assess clustering by subdistrict. Analyses were conducted using SAS9.2 software (SAS Institute, Cary, NC).

### Ethical approval

The study protocol was approved by both the Institutional Review Board at Guangdong Provincial Skin Diseases and STIs Control Center (Ref.5-39593) and the University of North Carolina at Chapel Hill (Ref.12-2457). All participants signed informed consent forms for participating in the study.

## Results

Out of the 1215 attempted surveys, 14 were partial completions, 368 were refusals, and 82 were non-responses, resulting in 751 full completions and a response rate was 62%, which is favourable for household surveys conducted in Mainland China [44]. The sample used in this paper included only participants reporting their HIV testing history. There were 85 participants who did not to answer questions related to HIV testing, resulting in 666 persons were included; 666 surveys included 323 (48.5%) at 18 subdistricts in Yuexiu district and 343 (51.5%) at 21 subdistricts in Tianhe district, respectively.

### Characteristics study subdistricts and participants

Characteristics varied among 39 subdistricts (Table 1). The mean driving distance from the participant’s residence to the 10 testing sites was 6.4 (SD = 1.1) kilometres in Yuexiu district and 12.4 (SD = 2.2) kilometres in Tianhe district (*P*<0.001). Differences in the proportion of men, 15–64 olds, unmarried, and migrants between Yuexiu and Tianhe districts were also statistically significant (*P*<0.05).

**Table 1:** Characteristics of study subdistricts, Guangzhou, China, 2014 (mean (SD)).

Characteristics	Yuexiu District *n* = 18	Tianhe District *n* = 21	Total *n* = 39	*t*-value	*P*-value
The total population (1000)	55.8(25.6)	68.2(45.0)	62.5(37.4)	-1.031	0.309
The proportion of men (%)	49.7(1.7)	53.1(2.7)	51.5(2.9)	-4.709	<0.001
The proportion of 15–64 years old (%)	77.6(2.9)	84.0(5.0)	81.0(5.3)	-5.039	<0.001
The proportion of college graduates (%)	33.0(11.4)	38.8(15.0)	36.1(13.6)	-1.331	0.191
The proportion of unmarried (%)	27.2(4.7)	39.4(14.7)	33.8(12.7)	-3.609	0.001
The proportion of employment (%)	52.9(6.5)	59.2(12.8)	56.3(10.7)	-1.962	0.059
The proportion of migrants (%)	30.2(13.7)	57.6(15.7)	44.9(20.1)	-5.814	<0.001
The number of health institutions per 1000 population	3.1(1.7)	3.1(1.2)	3.1(1.4)	- 0.143	0.887
The number of entertainment venues per 1000 population	3.0(2.2)	2.7(2.2)	2.9(2.2)	0.411	0.683
The mean driving distance from the participant’s residence to the 10 testing sites (kilometres)	6.4(1.1)	12.4(4.8)	9.5(4.6)	-22.321	<0.001

The mean age of participants was 32.7 (SD = 10.8). Almost half of the participants (48.8%) were female; more than half were married or cohabitating (62.4%), and about one-fourth (24.3%) had risky sexual behaviours in the past 12 months preceding the survey. The average social support score among participants was 38.1 (SD = 8.3). Bivariate analyses showed that differences in age, sex, social support, marital status, migration status, duration of living in Guangzhou, sexual status in the past 12 months, and district of residence between ever-tested and never-tested participants were statistically significant (*P*<0.05). Characteristics of participants were enumerated in Table 2.

### HIV testing status and location

Six hundred and sixty-six participants reported their HIV testing status of which 168 (25.2%, 95%CI: 21.9%, 28.5%) have ever been tested for HIV. The percentage of female and male participants who have ever been tested for HIV was 29.1% (99/340, 95%CI: 24.3%, 34.0%), and 21.2% (69/326, 95%CI: 16.7%, 25.6%), respectively. The percentage of persons who have ever been tested for HIV among individuals who reported risky sexual behaviours in the past 12 months preceding the survey was 26.5% (43/162, 95%CI:19.7%, 33.4%), which was not significantly different from the percentage of individuals who did not have risky sexual behaviours (125/504, 24.8%, 95%CI: 21.0%, 28.6%) (Table 2).

**Table 2:** Characteristics of study participants by HIV testing status, Guangzhou, China, 2014-resluts of bivariate analyses.

Characteristics	Ever-tested group *n* = 168	Never-tested group *n* = 498	Total *n* = 666	*t*-value/χ^2^	*P*-value
**Age (yrs)** mean (SD)	35.1(10.3)	31.8(10.8)	32.6(10.8)	-3.472	0.001
**Monthly personal income** median(interquartile range)	4000.0(2500.0–6000.0)	3000.0(1500.0–6000.0)	3300.0 (2000.0–6000.0)	-1.230	0.219
**Social support** mean (SD)	40.9(7.0)	37.0(8.3)	38.0(8.2)	-5.835	<0.001
**The mean driving distance from the participant’s residence to the 10 testing sites (Kilometers)** mean (SD)	9.2(4.7)	9.5(4.6)	9.5(4.6)	0.887	0.375
**Sex** *n* (%)					
Male	69(41.1)	257(51.7)	326(48.9)	5.579	0.020
Female	99(58.9)	241(48.4)	340(51.1)		
**Marital status** *n* (%)^#^					
Married/ Cohabiting	133(79.6)	280(57.0)	413(62.8)	27.269	<0.001
Single	34(20.4)	211(43.0)	245(37.2)		
**Education status** *n* (%)					
Primary school or below	9(5.4)	26(5.2)	35(5.3)	1.067	0.587
Secondary/High school	57(33.9)	191(38.4)	248(37.2)		
College or above	102(60.7)	281(56.4)	383(57.5)		
**Employment status** *n* (%)^&^					
Employed	133(81.1)	376(79.8)	509(80.2)	0.151	0.937
Unemployed	23(14.0)	69(14.6)	92(14.5)		
Others	8(4.9)	26(5.5)	34(5.4)		
**Migration status** *n* (%)					
Yes	83(49.4)	296(59.4)	379(56.9)	5.156	0.025
No	85(50.6)	202(40.6)	287(43.1)		
**Duration of living in Guangzhou** *n* (%)					
Less than 1 year	15(8.9)	64(12.9)	79(11.9)	13.013	0.002
1–5 years	30(17.9)	147(29.5)	177(26.6)		
More than 5 years	123(73.2)	287(57.6)	410(61.6)		
**Duration of living in current subdistrict** *n* (%)					
Less than 1 year	40(23.8)	152(30.5)	192(28.8)	4.455	0.107
1–5 years	53(31.5)	166(33.3)	219(32.9)		
More than 5 years	75(44.6)	180(36.1)	255(38.3)		
**Current sexual status** *n* (%)^#^ ^&^					
Yes	108(67.9)	224(51.5)	332(55.9)	12.750	<0.001
No	51(32.1)	211(48.5)	262(44.1)		
**Risky sexual behaviors in the past 12 months** *n* (%)					
Yes	43(25.6)	119(23.9)	162(24.3)	0.197	0.678
No	125(74.4)	379(76.1)	504(75.7)		
**Alcohol use** *n* (%)					
Harmful level or High-risk drinking	1(0.6)	8(1.6)	9(1.4)	3.165	0.367
Hazardous level	13(7.7)	41(8.2)	54(8.1)		
Low-risk drinking	140(83.3)	388(77.9)	528(79.3)		
Non-drinks	14(8.3)	61(12.2)	75(11.3)		
**District of residence** *n* (%)					
Yuexiu	101(60.1)	222(44.6)	323(48.5)	12.147	0.001
Tianhe	67(39.9)	276(55.4)	343(51.5)		

^#^: Eight and 65 participants refused to answer their marital status and sexual relationships, respectively.

^&^: Missing data for employment status, and sexual relationships in the past 12 months were 31 and5 cases.

**Fig 1:**
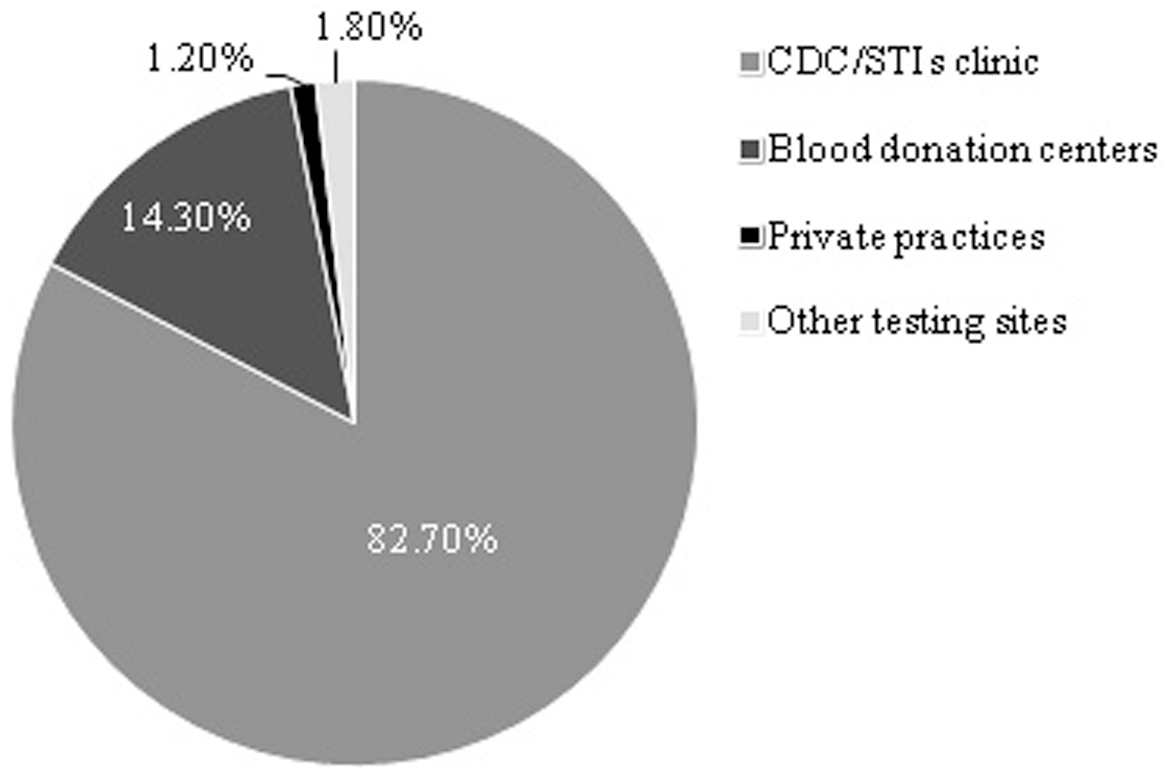
HIV testing location of participants, Guangzhou, China, 2014.

CDC/STIs clinics were the most popular testing sites (82.7%), followed by blood donation centres (14.3%), private practices (1.2%), and other testing sites (1.8%) (Fig 1). None of the participants reported using self-testing before.

### Spatial clusters of participants have ever been tested for HIV

**Fig 2:**
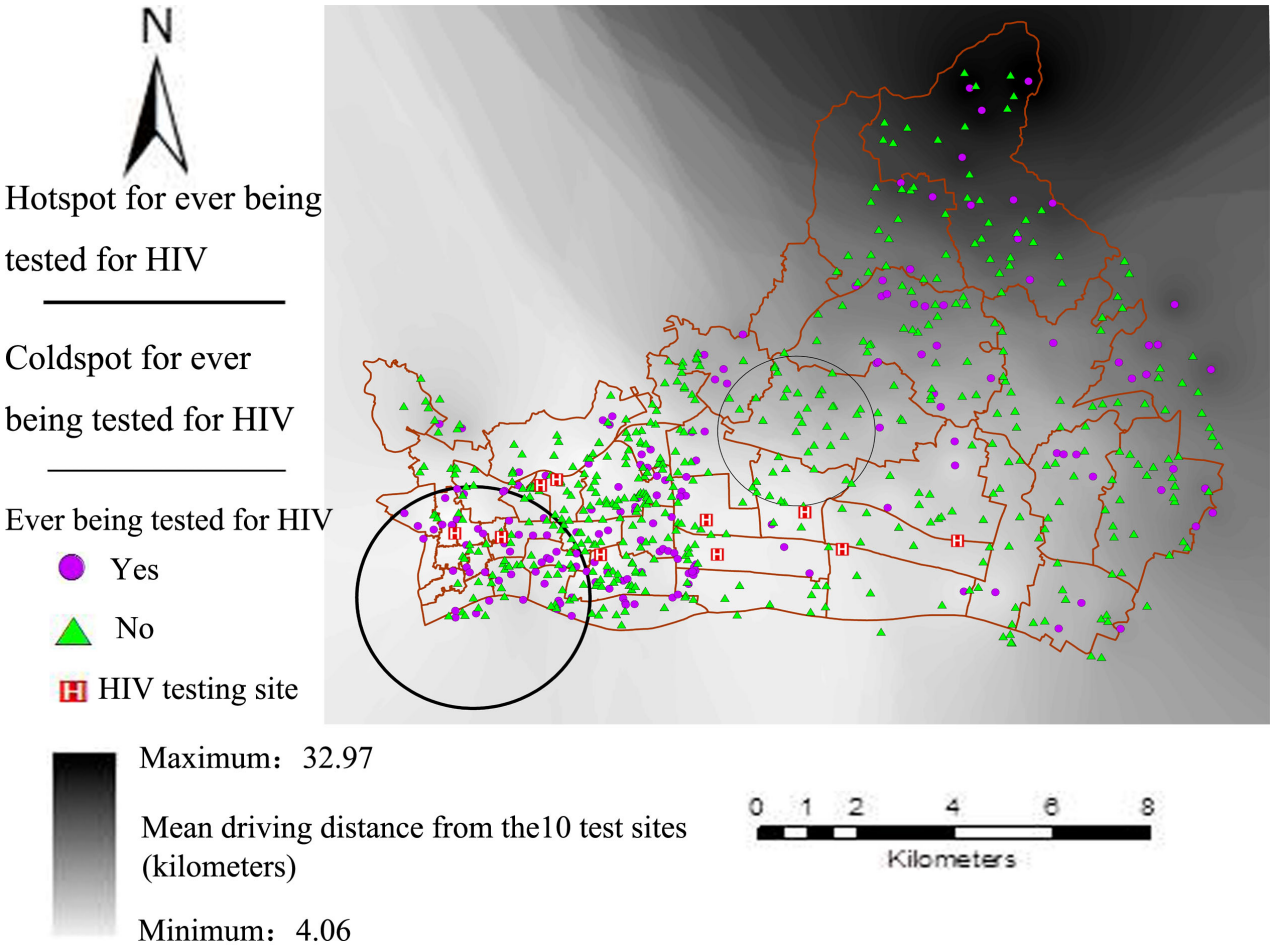
Spatial clustering of ever being tested for HIV and location of HIV testing sites within Yuexiu and Tianhe districts, Guangzhou, 2014.

Spatial cluster analyses found that there were a hotspot and a coldspot for ever being tested for HIV that located in Yuexiu and Tianhe districts, respectively (Fig 2). The hotspot included 48 study participants who have ever been tested for HIV and 25.8 expected cases (Rate Ratio = 1.86, *P* = 0.002), and the coldspot did not include observed participants while there were 9.8 expected cases (Rate Ratio = 0, *P* = 0.005). Fig 2 also showed the geographical distribution in HIV testing facilities, all facilities are concentrated in the downtown area in both districts. Differences in characteristics among participants residing in different clusters were listed in S1 Table.

### Factors associated with ever being tested for HIV

Two-level logistic regression models showed the associations between ever being tested for HIV and factors at both participant- and subdistrict-levels (Table 3). ICC for ever being tested for HIV was 13.6%, indicating that 13.6% of the variance in HIV testing uptake was accounted for by subdistrict-level factors. After controlling all significant confounding variables found in bivariate analyses (Table 2 and S2 Table), at the participant-level, a greater average driving distance from the participant’s residence to the 10 testing sites (aOR = 0.90, 95%CI: 0.84, 0.96) was significantly associated with lower likelihood of ever being tested for HIV. In other words, as the average driving distance increase by one kilometre, the odds of ever being tested for HIV decrease by 10%. Married or cohabiting participants (aOR = 2.14, 95%CI: 1.09, 4.20) and those with greater social support (aOR = 1.04, 95%CI: 1.01, 1.07) were more likely to be tested for HIV. No subdistrict-level factor was found being significantly associated with ever being tested for HIV.

**Table 3:** Two-level logistic regression analysis odds ratio and 95% CI for the association between geographic distance and ever being tested for HIV, Guangzhou, China, 2014.

Variable/Item	uOR	95%CI	*P*-value	aOR	95%CI	*P*-value
**Fixed effect**						
***Level-1(subdistrict)***						
The proportion of migrants	0.98	(0.97, 0.99)	0.001	0.98	(0.96, 1.01)	0.240
The proportion of unmarried	0.98	(0.96, 0.99)	0.002	0.97	(0.93, 1.01)	0.174
The proportion of 15–64 years old	0.94	(0.90, 0.98)	0.001	1.06	(0.89, 1.25)	0.514
***Level-2 (participant)***						
The mean driving distance from the participant’s residence to the 10 testing sites (Km)	0.99	(0.95, 1.04)	0.811	0.90	(0.84, 0.96)	0.001
Social support	1.06	(1.04, 1.09)	<0.001	1.04	(1.01, 1.07)	0.007
Age (yrs)	1.03	(1.01, 1.04)	0.002	0.99	(0.96, 1.02)	0.452
Sex						
Female	1.52	(1.06, 2.18)	0.022	1.29	(0.86, 1.94)	0.214
Male (Ref.)	1	-		1	-	
Marital status						
Married/ Cohabiting	2.87	(1.89, 4.37)	<0.001	2.14	(1.09, 4.20)	0.027
Single (Ref.)	1	-		1	-	
Migration status						
Yes	0.71	(0.49, 1.02)	0.063	0.98	(0.61, 1.57)	0.920
No (Ref.)	1	-		1	-	
Duration of living in Guangzhou						
Less than 1 year	0.57	(0.31, 1.05)	0.070	0.96	(0.44, 2.08)	0.909
1–5 years	0.50	(0.32, 0.78)	0.003	0.69	(0.39, 1.21)	0.193
More than 5 years (Ref.)	1	-		1	-	
Current sexual status						
Yes	1.96	(1.33, 2.89)	0.001	1.11	(0.57, 1.76)	0.642
No (Ref.)	1	-		1	-	
District of residence						
Yuexiu	1.91	(1.28, 2.85)	0.002	1.83	(0.96, 3.49)	0.067
Tianhe (Ref.)	1	-		1	-	
**Random effect**						
Variance of Level-2(σ^2^_*u*0_)			0.157			
Variance of Level-1(σ^2^_*e*_)			1.000			
***ICC* (%)**			13.57			

Abbreviations: uOR = unadjusted odds ratio; aOR = adjusted odds ratio; 95%CI = 95% confidence interval; ICC = intra-class correlation coefficient; Km = kilometres; Ref = reference.

## Discussion

HIV testing is essential to achieve the goal that 90% of people living with HIV knowing their HIV status [14] and our data show the percentage of participants who have ever been tested for HIV was 25.2% (95%CI: 21.9%, 28.5%), female participants reported a higher percentage (29.1%, 95%CI: 24.3%, 34.0%) than males (21.2%, 95%CI: 16.7%, 25.6%). The HIV testing rates were lower than self-reported figures found in other studies of the general population conducted in the United States (the overall percentage of ever being tested was 45%, and 50% of females and 40% of males) [45], Italy (32.8% of women, and 27.0% of men) [28], Kenya (34%) [32], and Botswana (48%) [31]. Different HIV testing policies in other countries, such as expanding HIV testing into community and nonclinical settings [46,47], and high HIV prevalence in African countries [48] may contribute to the difference.

The majority of the research on HTS have targeted key populations at higher risk of HIV infection [46,49]. Data on the general population’s HIV testing behaviours and barriers and facilitators to testing, especially geographic factors, remain scarce. This study shows that individuals have been tested for HIV concentrated in areas having testing sites nearby, and the greater the distance the lesser likelihood of the uptake of HTS in the general population. The findings underscore the importance of decentralizing facility-based HTS to increase access to the services and close the HIV testing gap by 2020 [14]. Studies examining the association between geographic distance and the uptake of HIV testing have produced inconsistent findings depending on the settings in which they were carried out. Research conducted in high-income countries has found an inverse relationship between geographic distance and the uptake of HIV testing, and this relationship was more pronounced among persons with lower income [30,50,51]. These findings are consistent with the current study. On the contrary, one study in Kenya showed distance did not appear to be a barrier to uptake of HIV testing after adjusting demographic characteristics. Individual’s perception of the importance of health care and HIV knowledge were major impact factors [32]. Two possible explanations are, first, the generalized HIV epidemic in Kenya [52] may contribute to more widespread HIV testing uptake, regardless of distance to testing site. Secondly, better HIV/AIDS literacy in more developed countries/regions may weaken the effect of knowledge. For example, less than 50% of young people in Kenya [52], and 90%-100% of American and Chinese undergraduates had comprehensive HIV knowledge [53,54]. We did not collect data on HIV perceptions due to low HIV prevalence in China [21].

In addition, the present study found that when residents in Guangzhou do test for HIV, 82.7% tested at CDC/STIs clinics. However, these government designated testing facilities are concentrated in the downtown area, and access to HIV testing services was inadequate in more rural or suburban divides. Our findings also demonstrated that none of the participants used self-testing. This is surprising given that HIV self-testing has been reported to be common among MSM in China [18], and may be due to low awareness of HIV self-testing among the general population. Therefore, our data also suggest there is a need for introducing non-facility based HTS to the general population. As WHO-recommended effective practices [1,15], community-based HTS, such as home-based, mobile outreach, and testing in schools, has been piloted in China to increase access to and coverage of HIV testing. For example, MSM and university students could purchase HIV self-testing kits via online services, community-based organizations, pharmacies, on-campus vending machines, and so on [18,19,55]. The spatial analysis could be a useful tool for scanning and locating areas lacking testing facilities and concentrations of populations with need for HTS and, therefore, be an instrumental tool for planning and implementing community-based HTS in the future. In addition, it is essential to further monitor and evaluate post-testing behaviours, linkage to care, as well as social harm and other adverse events in community-based HTS, especially HIV self-testing.

Individuals who have ever utilized HIV testing services were more likely to be married or have a stable partner, and have greater social support, which is consistent with previous studies [31,32], suggesting couples and partners, as well as supportive social networks, may play important roles in facilitating HTS’ utilization. People living with HIV or people perceived as high risk for infection may encourage their partners to be tested and provide mutual support. However, according to the WHO HIV Country Intelligence Database, in most countries, the proportion of couples and partners who test together is less than 20% [56]. Therefore, HIV partner notification services should also be promoted, because it has been shown that the service can increase HIV testing and lead to a stronger uptake of and adherence to HIV treatment and other prevention interventions [1,56].

This study is one of the first efforts to explore the association between geographic distance and the uptake of HIV testing from a representative population-based sample in China. However, our study has limitations. First, the information on HIV testing history was missing for 11% (85) of respondents in the present survey and it is possible that they may have different HIV testing characteristics. The non-response may be due to participants’ privacy concerns and fears of stigma and discrimination. In order to maximally reduce bias, we used tablet devices to conduct the interviews. The respondents read and responded privately, and their data was uploaded to a secure server cloud immediately by respondents themselves when the interviews were finished. Interviewers were not able to review the responses. To address possible bias, we examined differences in sociodemographic characteristics and risky sexual behaviours between participants did or did not report their testing status and no statistical differences were found, suggesting two groups shared similar characteristics (S3 Table). Second, a very small number of individuals reported HIV testing at non-CDC/STIs testing sites, which limited the analysis of factors associated with the choice of testing locations. Third, we did not collect data on awareness of HIV status of participants, which could contribute to establishing a more comprehensive understanding of HIV testing services in China. Future research is therefore needed to answer these questions. Fourth, driving distance used in this study did not take into account traffic condition; therefore the distance may not reflect the exact physical accessibility to HTS. In metropolitan cities, e.g. Guangzhou, strong public transportation infrastructure provides easy transportation within the city; however, people could also spend a lot of time waiting in public transportation or driving due to traffic congestion. Fifth, this was a cross-sectional study, therefore causality cannot be determined. Furthermore, it was confined to only one city in China. This may limit the generalizability of our results to other locations with different HIV epidemiological profiles and testing policies.

## Conclusions

Using population-representative survey data of adults in Guangzhou, China, we found that greater geographic distance was associated with lower likelihood of HTS utilization in the general population. In addition, couples and partners may play an important role in facilitating utilization of HTS. The results inform future HIV testing services planning and delivery in China and elsewhere with similar HIV epidemiological context and testing policies to increase access to and coverage of HTS and reach the first goal of the UNAIDS 90-90-90 target.

## Supporting information

S1 Table Characteristics of the participants within hotsopot and coldspot as compared with those residing outside of the clusters, Guangzhou, China, 2014. (DOCX)

S2 Table Results of bivariate two-level logistic regression model at the subdistrict-level, Guangzhou, China, 2014. (DOCX)

S3 Table Characteristics of study participants by response status, Guangzhou, China, 2014. (DOCX)

S1 File Minimal dataset. (XLS)

S2 File Questionnaires. (ZIP)
