# Supplementary material for: Is there a relationship between geographic distance and uptake of HIV testing services? A representative population-based study of Chinese adults in Guangzhou, China
Source: PLoS One. 2017 Jul 20;12(7):e0180801. doi: 10.1371/journal.pone.0180801 (PMC5519047; doi:10.1371/journal.pone.0180801)
Supplement: S1 Table — (DOCX) [file pone.0180801.s001.docx]

**S1 Table Characteristics of the participants within hotsopot and coldspot as compared with those residing outside of the clusters, Guangzhou, China, 2014.**

| **Characteristics** | **Hotspot**  ***n*=103** | **Coldspot**  ***n*=39** | **Outside of clusters**  ***n*=524** | ***P*-value** |
| --- | --- | --- | --- | --- |
| **Age (yrs)** mean(SD) | 37.7(11.1) | 30.3(10.7) | 31.8(10.5) | <0.001 |
| **Monthly personal income**  median(interquartile range) | 3500(2000-5650) | 2000(0-5000) | 3500(2000-6000) | 0.088 |
| **Social support** mean(SD) | 39.6(8.3) | 36.1(7.7) | 37.8(8.2) | 0.045 |
| **Mean driving distances from the 10 test sites (kilometers)** mean(SD) | 6.7(0.9) | 8.3(1.5) | 10.1(5.0) | <0.001 |
| **Sex** *n* (%) |  |  |  | 0.020 |
| Male | 39(37.9) | 16(41.0) | 271(51.7) |  |
| Female | 64(62.1) | 23(59.0) | 253(48.3) |  |
| **Marital status** *n* (%)^#^ |  |  |  | 0.003 |
| Married/ Cohabiting | 78(76.5) | 19(50.0) | 316(61.0) |  |
| Single | 24(23.5) | 19(50.0) | 202 (39.0) |  |
| **Education status** *n* (%) |  |  |  | 0.282 |
| Primary school or below | 8(7.8) | 0(0.0) | 27(5.2) |  |
| Secondary/High school | 40(38.8) | 12(30.8) | 196(37.4) |  |
| College or above | 55(53.4) | 27(69.7) | 301(57.4) |  |
| **Employment status** *n* (%)^&^ |  |  |  | 0.028 |
| Employed | 74(73.3) | 23(67.6) | 412(82.4) |  |
| Unemployed | 17(16.8) | 9(26.5) | 66(13.2) |  |
| Others | 10(9.9) | 2(5.9) | 22(4.4) |  |
| **Migration status** *n* (%) |  |  |  | <0.001 |
| Yes | 36(35.0) | 21(53.8) | 322(61.5) |  |
| No | 67(65.0) | 18(46.2) | 202 (38.5) |  |
| **Duration of living in Guangzhou** *n* (%) |  |  |  | <0.001 |
| Less than 1 year | 4(3.9) | 3(7.7) | 72(13.7) |  |
| 1-5 years | 16(15.5) | 17(43.6) | 144(27.5) |  |
| More than 5 years | 83(80.6) | 19(48.7) | 308(58.8) |  |
| **Duration of living in current subdistrict** *n* (%) |  |  |  | <0.001 |
| Less than 1 year | 17(16.5) | 9(23.1) | 166(31.7) |  |
| 1-5 years | 31(30.1) | 21(53.8) | 167 (31.9) |  |
| More than 5 years | 55(53.4) | 9 (23.1) | 191(36.5) |  |
| **Current sexual status** *n* (%)^# &^ |  |  |  | 0.083 |
| Yes | 58(62.4) | 16(41.0) | 258(55.8) |  |
| No | 35(37.6) | 23(59.0) | 204(44.2) |  |
| **Risky sexual behaviors in the past 12 months** *n* (%) |  |  |  | 0.985 |
| Yes | 25(24.3) | 10(25.6) | 127(24.2) |  |
| No | 78(75.7) | 29(74.4) | 397(75.8) |  |
| **Alcohol use** *n* (%) |  |  |  | 0.646 |
| Harmful level or High-risk drinking | 1(1.0) | 1(2.6) | 7(1.3) |  |
| Hazardous level | 6(5.8) | 1(2.6) | 47(9.0) |  |
| Low-risk drinking | 86(83.5) | 32(82.0) | 410(78.2) |  |
| Non-drinks | 10(9.7) | 5(12.8) | 60(11.5) |  |
| **District of residence** *n* (%) |  |  |  | <0.001 |
| Yuexiu | 103(100.0) | 0(0.0) | 220(42.0) |  |
| Tianhe | 0(0.0) | 39(100.0) | 304(58.0) |  |

^#^: Eight and 65 participants refused to answer their marital status and sexual relationships, respectively.

^&^: Missing data for employment status, and sexual relationships in the past 12 months were 31 and5 cases.
