# Supplementary material for: Is there a relationship between geographic distance and uptake of HIV testing services? A representative population-based study of Chinese adults in Guangzhou, China
Source: PLoS One. 2017 Jul 20;12(7):e0180801. doi: 10.1371/journal.pone.0180801 (PMC5519047; doi:10.1371/journal.pone.0180801)
Supplement: S2 Table — (DOCX) [file pone.0180801.s002.docx]

**S2 Table Results of bivariate two-level logistic regression model at the subdistrict-level, Guangzhou, China, 2014**

| **Variable/Item** | ***uOR*** | ***95%CI*** | | ***t-value*** | ***P-value*** |
| --- | --- | --- | --- | --- | --- |
| **Fixed effect** |  |  | |  |  |
| The proportion of men (%) | 0.93 | 0.86,1.00 | | -1.953 | 0.051 |
| The proportion of 15-64 years old (%) | 0.94 | 0.90,0.98 | | -3.199 | **0.001** |
| The proportion of college graduates (%) | 0.99 | 0.98,1.01 | | -0.981 | 0.327 |
| The proportion of unmarried (%) | 0.98 | 0.96,0.99 | | -3.055 | **0.002** |
| The proportion of employment (%) | 0.99 | 0.97,1.01 | | -0.888 | 0.375 |
| The proportion of migrants (%) | 0.98 | 0.97,0.99 | | -3.239 | **0.001** |
| The number of health institutions per 1000 population | 0.94 | 0.81,1.10 | | -0.734 | 0.463 |
| The number of entertainment venues per 1000 population | 1.02 | 0.91,1.14 | | 0.288 | 0.773 |
|  |  |  | |  |  |
| **Random effect (estimates*)*** |  |  |  | |  |
| *Level-2*(σ^2^*_u_*_0_) | 0.157 | 0.117 |  | |  |
| *Level-1*(σ^2^*_e_*) | 1.000 | 0.000 | - | | - |

Abbreviations: uOR=unadjusted odds ratio; 95%CI=95% confidence interval.
