# Supplementary material for: Is there a relationship between geographic distance and uptake of HIV testing services? A representative population-based study of Chinese adults in Guangzhou, China
Source: PLoS One. 2017 Jul 20;12(7):e0180801. doi: 10.1371/journal.pone.0180801 (PMC5519047; doi:10.1371/journal.pone.0180801)
Supplement: S3 Table — (DOCX) [file pone.0180801.s003.docx]

**S3 Table Characteristics of study participants by response status, Guangzhou, China, 2014**

| **Characteristics** | **Reported testing status group**  ***n*=666** | **Non-reported group**  ***n*=85** |
| --- | --- | --- |
| **Age (yrs)** mean(SD) | 32.6(10.8) | 34.6(11.6) |
| **Monthly personal income (RMB)**  median(interquartile range) | 3300.0 (2000.0-6000.0) | 3250.0(2050.0-5000.0) |
| **Social support** mean(SD) | 38.0(8.2) | 38.6(8.0) |
| **Sex** *n* (%) |  |  |
| Male | 326(48.9) | 47(44.7) |
| Female | 340(51.1) | 38(55.3) |
| **Marital status** *n* (%) |  |  |
| Married/ Cohabiting | 413(62.8) | 55(66.3) |
| Single | 245(37.2) | 28(33.7) |
| **Education status** *n* (%) |  |  |
| Primary school or below | 35(5.3) | 5(6.0) |
| Secondary/High school | 248(37.2) | 39(46.4) |
| College or above | 383(57.5) | 40(47.6) |
| **Employment status** *n* (%) |  |  |
| Employed | 509(80.2) | 69(85.2) |
| Unemployed | 92(14.5) | 7(8.6) |
| Others | 34(5.4) | 5(6.2) |
| **Migration status** *n* (%) |  |  |
| Yes | 379(56.9) | 53(56.9) |
| No | 287(43.1) | 32(43.1) |
| **Duration of living in Guangzhou** *n* (%) |  |  |
| Less than 1 year | 79(11.9) | 13(15.3) |
| 1-5 years | 177(26.6) | 16(18.8) |
| More than 5 years | 410(61.6) | 56(65.9) |
| **Current sexual status** *n* (%) |  |  |
| Yes | 332(55.9) | 35(61.4) |
| No | 262(44.1) | 22(38.6) |
| **Risky sexual behaviors in the past 12 months** *n* (%) |  |  |
| Yes | 162(24.3) | 26(30.6) |
| No | 504(75.7) | 59(69.4) |
| **Alcohol use** *n* (%) |  |  |
| Harmful level or High-risk drinking | 9(1.4) | 3(3.5) |
| Hazardous level | 54(8.1) | 6(7.1) |
| Low-risk drinking | 528(79.3) | 66(77.6) |
| Non-drinks | 75(11.3) | 10(1.8) |
