# Supplementary material for: Is there a relationship between geographic distance and uptake of HIV testing services? A representative population-based study of Chinese adults in Guangzhou, China
Source: PLoS One. 2017 Jul 20;12(7):e0180801. doi: 10.1371/journal.pone.0180801 (PMC5519047; doi:10.1371/journal.pone.0180801)
Supplement: S2 File — (ZIP) [file pone.0180801.s005.zip › questionnaire/Questionnaire -English version.pdf]

Guangzhou residents health status survey

Inclusion criteria:
Which year were you born?_ _______
(Exclude those born out of the range 1965-1996)

PARTICIPANT'S I.D. NUMBER

__ __ __ _____

ENTER TODAY'S DATE

__ __ / __ __ / __ __ __ __mm / dd / yyyy

INTERVIEWER I.D. NUMBER

__ __ __

BACKGROUND/DEMOGRAPHICS (B) (18 ITEMS)

B1. What is your biological sex?

1. Male
2. Female

B2. What is your ethnicity?

1. Han Chinese
2. Other ethnic minority Please specify__________.

B3. What grade did you complete in school?

0. Never attended school
1. Elementary school
2. Junior high school
3. Senior high school/vocational high school/professional training school
4. Associate degree
5. College degree or higher
99. Refuse to answer

B4. How would you describe your current relationship status?

0. Never married nor cohabitating
1. cohabitating but not married
2. Married
3. Divorced
4. Widowed

99. Refuse to answer

B5. What best describes your current living situation?

1. Live in a house or apartment that I own
2. Live in a house or an apartment I rent
3. Rent a room or space in someone else's house or apartment
4. Stay with someone else for free
5. Other (Specify ____________________)

99. Refuse to answer

B6. Are you currently living? (allow multiple choices)

1. By yourself
2. With co-workers
3. With friends/people from your hometown
4. With spouse/lover
5. With parents/in-laws
6. With kids
7. Other (Specify ____________________)

99. Refuse to answer

B7. Most of the time, how many other people live in the same household/place with you?__________

B8. How long have you lived in your current neighborhood?

1. 0~3 months
2. 4~6 months
3. 7 months ~1 year
4. 1~5 years
5. >5 years

B9. How long have you lived in Guangzhou?

1. 0~3 months
2. 4~6 months
3. 7 months ~1 year
4. 1~5 years
5. >5 years

B10. Do you have permanent household registration (i.e., *Hukou*) in Guangzhou?

1. No
2. Yes

B11. Where were you born? __________city __________county **(if Guangzhou City, skip to B13)**

B12. What is the purpose that you moved here to Guangzhou?

1. For work
2. For joining family
3. For study
4. Others, please specify.______ ___

B13. How many locations (this includes cities, townships, small rural villages) have you lived for more than 3 months in the past 5 years. ________

B14. Which was the last location you lived before GZ? ________

B15. What is your current employment status? Are you:
1. Employed full time
2. Employed part time
3. Unemployed but seeking work **(skip to B17)**
4. Unemployed - not seeking work **(skip to B17)**
5. Work for yourself
6. Other (Specify ______________________)
77. Don't Know
88. Not Applicable
99. Refuse to Answer

B16. How would you describe what you do in your current job?
1. Agricultural producer [farmer]
2. Manual worker
3. Sales, service, entertainment industry worker
4. Self-employed, independent worker [geti hu/laodong zhe, < 8 workers]
5. Clerical worker, low-rank bureaucrat, office worker
6. Technical worker, teacher, intellectual [professional/technical worker]
7. Manager, factory director, business-owner (including internet shops, soho)
8. Government official (including village official)
9. Other occupation, specify____________

B17. In the past 30 days, how much money did you earn altogether in RMB?
____

B18. In the past 30 days, how much money did your family earn altogether in RMB?
____

Now we ask about your health related questions.

GENERAL PHYSICAL HEALTH (G) (5 ITEMS)

G1. In general, would you say your health is:
1. Excellent
2. Very good

3. Good
4. Fair
5. Poor

G2. What is your height? ________ centimeters

G3. What is your weight? ________ kilograms

G4. Have you been told by a doctor or other health professional that you have any physical illnesses in the past 12 months?

0. No (skip to the next section)

1. Yes

G5. What the illnesses were you were diagnosed with? _________

(allow for multiple choices)

1. Back ache lasting for 3 months or longer
2. Any other muscle or bone disease lasting for 3 months or longer
3. Any other neurological condition, apart from Parkinson's disease and epilepsy
4. Cancer
5. Any thyroid condition
6. Any (ovarian/testicular) or pituitary condition
7. WOMEN ONLY: I have received IVF or other fertility treatment
8. Others, please specify. __________

HEALTH BEHAVIORS (HB) (29 ITEMS)

HB1. Thinking about just the past 30 days… out of the past 30 days, how many days did you smoke cigarettes? Your best estimate is fine. __________ (**RANGE: 0 to 30, If 0, skip HB3**)

HB2. In the past 30 days, on those days when you smoked, on average, how many cigarettes did you smoke per day? Your best estimate is fine and a pack = 20 cigarettes. ________ (cigarettes per day)

READ this introduction/instructions:

The following questions relate to your usual sleep habits during the past month only. Your answers should indicate the most accurate reply for the majority of days and nights in the past month.

HB3. During the past month, what time have you usually gone to bed at night (AM or PM)? ________
HB4. During the past month, how long (in minutes) has it usually taken you to fall asleep each night? _______
HB5. During the past month, what time have you usually gotten up in the morning (AM or PM)? _________

HB6. During the past month, how many hours of actual sleep did you get per night? (This may be different than the number of hours you spent in bed)? _________

HB7. Regarding the past month, how would you rate your sleep quality overall?

Very bad (1)   Fairly bad (2)   Fairly good (3)   Very good (4).

HB8. How much did these sleep problems interfere with:

		None	Mild	Moderate	Severe	Extreme or cannot do
**HB8a**	Maintaining social relationships?	1	2	3	4	5
**HB8b**	Your day-to-day work/school?	1	2	3	4	5

READ this introduction/instructions:

Now I am going to ask you some questions about your use of alcoholic beverages during this past year.

An alcoholic beverage is one 12oz beer, 5oz glass of wine, 1.5 once shot of hard alcohol or baijiu.

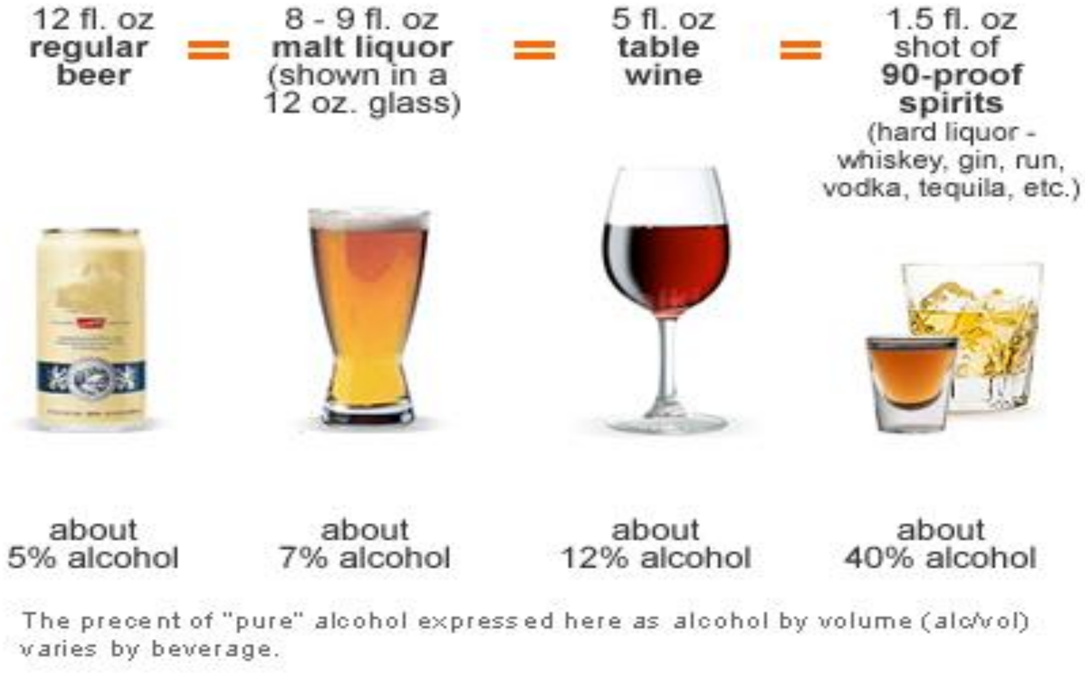

HB9. How often do you drink alcohol? (Choose one)

0. Never **（skip to the HB19）**

1. Monthly or less

2. 2-4 times a month

3. 2-3 times a week

4. 4 or more times a week

77. Don't Know
88. Not Applicable
99. Refuse to Answer

HB10. How many drinks containing alcohol do you have on a typical day when you are drinking? (Choose one)

1. 1 or 2
2. 3 or 4
3. 5 or 6
4. 7 to 9
5. 10 or more

77. Don't Know
88. Not Applicable
99. Refuse to Answer

HB11. How often do you have six or more drinks on one occasion? (Choose one)

0. Never
1. Less than monthly
2. Monthly
3. Weekly
4. Daily or almost daily

77. Don't Know
88. Not Applicable
99. Refuse to Answer

HB12. How often, during the past year, have you found that you were not able to stop drinking once you had started?

0. Never
1. Less than monthly
2. Monthly
3. Weekly
4. Daily or almost daily

77. Don't Know
88. Not Applicable
99. Refuse to Answer

HB13. How often, during the past year, have you failed to do what was normally expected of you because of drinking?

0. Never
1. Less than monthly
2. Monthly
3. Weekly
4. Daily or almost daily
77. Don't Know
88. Not Applicable
99. Refuse to Answer

HB14. How often, during the past year, have you needed a drink in the morning to get yourself going after a heavy drinking session?

0. Never
1. Less than monthly
2. Monthly
3. Weekly
4. Daily or almost daily
77. Don't Know
88. Not Applicable
99. Refuse to Answer

HB15. How often, during the past year, have you had a feeling of guilt or remorse after drinking?

0. Never
1. Less than monthly
2. Monthly
3. Weekly
4. Daily or almost daily
77. Don't Know
88. Not Applicable
99. Refuse to Answer

HB16. How often, during the past year, have you been unable to remember what happened the night before because you had been drinking?

0. Never
1. Less than monthly
2. Monthly
3. Weekly
4. Daily or almost daily
77. Don't Know

88. Not Applicable
99. Refuse to Answer

HB17. Have you or has someone else been injured as a result of your drinking?

0. No

1. Yes, but not in the past year

2. Yes, during the past year

77. Don't Know
88. Not Applicable
99. Refuse to Answer

HB18. Has a relative, friend, doctor, or other health worker been concerned about your drinking or suggested you cut down?

0. No

1. Yes, but not in the past year

2. Yes, during the past year

77. Don't Know
88. Not Applicable
99. Refuse to Answer

READ this introduction/instructions:

Now we would like to ask about drugs you have used.

HB19. Please indicate whether you have used any of the following substances in the past month, or if have ever used these substances. For drugs that you've used, we will also ask if you used them before or during sex.

**Drugs**	**Used in Past month?**	**Used before or during sex within the past month?**	**If no to use in past month: Have you ever used before or during sex**	**If yes to ever used: Have you ever used this drug before or during sex?**
1. Heroin	0	1	2	
2. Cocaine	0	1	2	

3. Methamphetamine	0	1	2	
4. Ketamine	0	1	2	
5. Morphine	0	1	2	
6. Cannabis	0	1	2	
7. Meperidine	0	1	2	
8. Buprenorphine	0	1	2	
9. Phencyclidine	0	1	2	
10. Alcohol	0	1	2	
11. Other, specify ______________	0	1	2	

systematic / original terms	trivial names IN CHINESE
**heroin**	白粉、白面儿、四号、四号仔、软仔、小仔
**cocaine**	可卡因、可可、快克、crack、snow
**Amphetamine / Methamphetamine**	猪肉、安公子、安仔、冰糖、冰块、盐、Speed、糖果、冰毒
**ketamine**	卡门、K、K 他命、Special K、K 粉、克他命、K 仔
**morphine**	魔啡
**Marijuana / Cannabis**	草、饭、麻仔、老鼠尾
**Meperidine / Pethidine**	杜冷丁、度冷丁、哌啶、配西汀、地美露（Demerol）、盐酸哌替啶
**buprenorphine**	布诺啡、叔丁啡、丁苯诺啡、Temegesic、Buprenex
**PCP(Phencyclidine)**:	天使尘、Love boat
**Ecstasy**	忘我、快乐丸、绿蝴蝶、亚当、狂喜、衣服、Eve、夏娃

MENTAL HEALTH (MH) (24 ITEMS)

MH1. Have you ever been told by a doctor or other health professional that you have any Psychological health problem?

1. Yes
0. No (skip to next section)
77. Don't Know
88. Refuse to Answer

MH2. What Psychological health problem did a doctor say that you might have? ________

MH3. Over the last 3 months, did you have any of the following problems?

**Problems**	**No**	**Yes**
1.Persistent and distressing complaints of increased fatigue after mental effort	0	1
2. Persistent and distressing complaints of bodily weakness and exhaustion after minimal effort	0	1
3. Feelings of muscular aches and pains - dizziness	0	1
4. Tension headaches	0	1
5. Sleep disturbance	0	1
6. Inability to relax – irritability	0	1
7. Dyspepsia (Indigestion)	0	1

MH4. How much did these symptoms interfere with:

		None	Mild	Moderate	Severe	Extreme or cannot do
**MH4a**	Maintaining social relationships?	1	2	3	4	5
**MH4b**	Your day-to-day work/school?	1	2	3	4	5

MH5. PHQ-9 (Depression):

Over the last 2 weeks, how often have you been bothered by any of the following problems?

	Not at all	Several days	More than half the days	Nearly every day
1. Little interest or pleasure in doing things	1	2	3	4
2. Feeling low, depressed or hopeless	1	2	3	4
3. Trouble falling or staying asleep or sleeping too much	1	2	3	4
4. Feeling tired or having little energy	1	2	3	4
5. Poor appetite or overeating	1	2	3	4
6. Feeling bad about yourself – or that you are a failure or have let yourself or your family down	1	2	3	4
7. Trouble concentrating on things, such as reading the newspaper or watching television	1	2	3	4
8. Moving or speaking so slowly that other people could have noticed. Or the opposite – being so fidgety or restless that you have been moving around a lot more than usual.	1	2	3	4

9. Thoughts that you would be better off dead, or of hurting yourself in some way	1	2	3	4

MH6. How much did these symptoms interfere with:

		None	Mild	Moderate	Severe	Extreme or cannot do
**MH6a**	Maintaining social relationships?	1	2	3	4	5
**MH6b**	Your day-to-day work/school?	1	2	3	4	5

MH7. In the past 12 months, did you seek the assistance of any of the following people or for help with your emotions or mental health: (can include multiple selections)

1. Never sought assistance **(skip to the next section.)**
2. Psychiatrist
3. Mental health counselor
4. Other physician
5. Social worker
6. Other people , specify ______________

MH8. What were you hoping to gain from seeking the assistance of [insert MH7]?

____

SOCIAL SUPPORT and COMMUNITY TRUST (SC) (14 items)

SC1. How many close friends that you can gain support and help from them?

1. None
2. 1 - 2
3. 3 - 5
4. 6 or above

SC2. This one year you: □

1. Lived alone
2. Stayed with strangers
3. Lived with classmates, colleagues or friends.
4. Lived with family

SC3. You and your neighbours: □

1. Not care each other
2. Might help a little bit if necessary
3. Some of them really care about you
4. Most of them really care about you

SC4. You and your colleagues: □

1. Not care each other
2. Might help a little bit if necessary
3. Some of them really care about you
4. Most of them really care about you

SC5. Support and Care from family members

	**Have this member or not**	**Support and Care from this member**			
		**None**	**Seldom**	**Normal**	**Highly**
		**0**	**1**	**2**	**3**
Partner	Yes/no				
Parents	Yes/no				
Children	Yes/no				
Siblings	Yes/no				
Other members	Yes/no				

SC6. When urgent, from whom you can seek for financial support and solution for problem?

① Financial support from no one

② Financial support from :**（multiple choice）**

1）Partner 2） Other family members 3）friends 4）relatives 5）colleagues

6）Job units 7） government 8）NGO

9）others（________________）

SC7. When urgent, from whom you can seek for care and comfort?

① Financial support from no one

② Financial support from :**（multiple choice）**

1）Partner 2） Other family members 3）friends 4）relatives 5）colleagues

6）Job units 7） government 8）NGO

9）others（________________）

SC8. Will you and how will you, if any, speak out your worries? □

1. Never
2. Yes. I will speak with 1 to 2 close friends of mine
3. Only when my friends ask, then I will speak it out
4. Yes. I will actively speak it out to gain support and understanding from others

SC9. Will you seek for help if you are in trouble? □

1. Never
2. Seldom
3. Sometimes
4. Always

SC10. Will you join society activities? □

1. Never
2. Yes. I will speak with 1 to 2 close friends of mine
3. Only when my friends ask, then I will speak it out
4. Yes. I will actively speak it out to gain support and understanding from others

Now we would like to ask about your opinion of people in your neighborhood.

SC11. 'Most people in this village/neighborhood are honest and can be trusted'. Do you agree this statement?
1. 'Strongly Disagree'
2. 'Disagree'
3. 'Agree'
4. 'Strongly Agree'

To ask migrants: (people who have GZ *hukou*)

SC12. 'I feel welcome here in Guangzhou by the people who live here.' Do you agree this statement?
1. 'Strongly Disagree'
2. 'Disagree'
3. 'Agree'
4. 'Strongly Agree'

To ask non-migrants (people who have GZ *hukou*)

SC13.'I believe that migrants from other provinces should not live in Guangzhou' Do you agree this statement?
1. 'Strongly Disagree'
2. 'Disagree'
3. 'Agree'
4. 'Strongly Agree'

SC14.'I believe that migrants from other provinces should not receive local hukou.' Do you agree this statement?
1. 'Strongly Disagree'
2. 'Disagree'
3. 'Agree'
4. 'Strongly Agree'

READ

Now we would like to ask about your sexual behavior and health.

SEX PARTNERS (S) (10 items)

S1. How would you describe your sexual orientation?

1 .Heterosexual

2. Homosexual

3. Bisexual

4. Unsure

S2. Up till now, have you ever had vaginal sex with anyone (including having had sex with a person only once)?

1. Yes, often

2. Yes, sometimes

3. Yes, Rarely

4. No, never

S3. Nowadays, many people have anal sex. Do you feel dirty about these things?

1. Yes
2. No

S4. Have you ever had anal sex with your sex partners (including only once)?

1. Yes, often

2. Yes, sometimes

3. Yes, Rarely

4. No, never **(skip to SECTION 'Sexually Transmitted Diseases')**

S5. Do you currently (during the last 12 months) have a sexual relationship with someone (including both vaginal and anal sex, and any kind of sex partner)?

1. Yes (go to S6)
2. No **(skip to SECTION 'Sexually Transmitted Diseases')**

S6. During the last 12 months, how many people do you have a sexual relationship with (including both vaginal and anal sex)?

1. 1
2. 2~3 **(skip to S8)**
3. 4~5 **(skip to S8)**

4. More than 5 **(skip to S8)**

S7. Do you and the person with whom you have sex currently live together?

1. Yes
2. No **(skip to SECTION 'Short Term Partner')**

S7a. Is this person a man or a woman?

1. Man **(skip to C1 (Introduction to Section C), then C2-1)**
2. Woman **(C1 (Introduction to Section B), then C2-2)**

S8. You mentioned that there is more than one person with whom you had sex; among them is there anyone lives with you together?

1. Yes
2. No **(skip to SECTION 'Short Term Partner')**

S8a. Is this person a man or a woman?

1. Man **(skip to C1 (Introduction to Section C), then C2-1)**
2. Woman **(C1 (Introduction to Section B), then C2-2)**

Current Partner (C) (9 items)

C1. Below we are going to ask about the person, with whom you have sex currently and live together with each other.

C2-1. Please pick a title for this man. Which of the following terms do you think is most appropriate for you to address this person? [Ask only if S6=1]

1. Spouse (airen/laogong)
2. Boyfriend (nan pengyou)
3. Secret lover (qingren)
4. Friend (shuren, pengyou)
5. Acquaintance (yibanpengyou)
6. Key pal (wangyou)
7. Boss (laoban)
8. Superior (shangji)
9. Business customers
10. Customer (guke) [implies respondent is a commercial sex worker and this was her client
11. Other

C2-2. Please pick a title for this woman. Which of the following terms do you think is most appropriate for you to address this person? [Ask only if S6=2]

1. Spouse (airen/laopo)
2. Girlfriend (nü pengyou)
3. Second wife (ernai)
4. Friend (shuren,pengyou)
5. Acquaintance (yibanpengyou)
6. Key pal (wangyou)
7. PR (Public Relations) girl (xiaojie) [literally, "miss," which implies "commercial sex worker"]
8. Other

C3. How long did this sexual relationship last?

1. Less than one month
2. 1~ 6 months
3. 7~12 months
2. Less than 2 years
3. 2 ~ 4 years
4. More than 4 years

C4. During the time of this sexual relationship, did you have sex with anyone other than this partner (count everyone, even if it happened just once)?

1. Yes
2. No

C5. At the time of this sexual relationship, did your partner have sex with anyone other than you (even if it happened just once)?

1. Yes, definitely
2. Perhaps, I don't know for sure
3. No, definitely

C6. When was the last time you had sex with this partner?

1. Within last one week
2. Within last two weeks
3. Within last one month
4. More than one month ago but within a year
5. More than a year ago **[skip to ST1]**

C7. During the last 12 months, at the time you had sexual relationship with this partner, how often did you have sex with each other?

1. Once a day or more
2. 3 to 6 times a week

3. Once or twice a week
4. 2 to 3 times a month
5. Once a month or less

C8. How often did you use a condom when having sex with this partner?

1. Always **[skip to ST1]**
2. Often
3. Rarely
4. Never **[skip to ST1]**

C9. Did you use a condom the last time you had sex with this partner?

1. Yes
2. No

Short Term Partner (ST) (12 items)

ST1. During the last four weeks, how many people do you have a sexual relationship with (including any kinds of sex partner, even long term partner)? _________

ST2. During the last four weeks, how many new sex partners did you have sex with for the first time ? (including any kinds of sex partner)? _________

ST3. Now we ask about the person with whom you had a sexual relationship most recently. Was the person a man or a woman?

1. Man **(skip to ST4)**
2. Woman **(skip to ST5)**

ST4. Please select a title among the following. Which of the followings do you think is the most appropriate term to address your partner? [Ask only if ST3=1]

1. Boyfriend (nan pengyou)
2. Fiancé (weihun fu)
3. Ex-husband (qianfu)
4. Secret lover (qingren)
5. Friend (shuren, pengyou)
6. Acquaintance (yibanpengyou)
7. Key pal (wangyou)
8. Boss (laoban)
9. Superior (shangji)
10. Business customers

11. Customer (guke) [implies respondent is a commercial sex worker and this was her client
12. Sex partner (xingban/xinghuoban/xingbanlv)
13. Other

ST5. Please select a title among the following. Which of the following terms do you think is most appropriate for you to address this person? [Ask only if ST3=2]

1. Girlfriend (nü pengyou)
2. Fiancée (weihunqi)
3. Second wife (ernai)
4. Friend (shuren,pengyou)
5. Acquaintance (yibanpengyou)
6. Key pal (wangyou)
7. PR (Public Relations) girl (xiaojie) [literally, "miss," which implies "commercial sex worker"]
8. Ex-wife (qianqi)
9. Sex partner (xingban/xinghuoban/xingbanlv)
10. Other

ST6. Up till now, how many times did you have sex with this partner?

________[If >1, skip to ST8.]

ST7. Did you use a condom when having sex with this partner that time? [Ask if had sex only once]

1. Yes **[skip to ST9]**
2. No **[skip to ST9]**

ST8. When having sex with this partner, how often did you use a condom?

1. Always
2. Often
3. Rarely
4. Never

ST9. How long ago did you have sex with this partner for the last time?

1. Within one week
2. Within two weeks
3. Within four weeks
4. More than four weeks ago (To remind the participants to think again because we are talking about the most recent partner within 4 weeks.)

ST10. How long the sexual relationship last between you and this partner?

1. Less than one week
2. Less than one month
3. 1~ 3 months
4. 4~6 months
5. 7~12 months
6. More than 1 year
7. More than 4 years

ST11. At the time of this sexual relationship, did you have sex with anyone other than this partner (count everyone, even if it happened just once)?

1. Yes
2. No

ST12. At the time of this sexual relationship, did this partner have sex with anyone other than you (even if it happened just once)?

1. Yes, definitely
2. Perhaps, I don't know for sure
3. No

Sexually Transmitted Diseases (D) (24 ITEMS)

D0. Up to this point of your life, how many people did you have sex with (even if you did it with someone only once)? Please include all people, including those you have divorced, separated or those deceased. Please also include people you had sex with who were of the same biological sex as you. _________

D1. In China today, many people are infected with sexually transmitted diseases (STDs, as list below), and they are infected with the diseases not just because of what they have done themselves. Up till now, have you ever been tested for following STDs?

STDs	**D1a.**I was never tested of this(skip to **the next row**)	**D1b.** Yes	**D1c*.** Where did you get the test from?	**D1d.**During your lifetime, have you had this? (if No=2, skip to **the next row**)	**D1e**.**How did you know you had this **?**	**D1f.**Times I was told I had this in the past 12 months	**D1g#.**When you had this last time, what did you do for treatment?
Chlamydia							
Gonorrhoea							
Genital Warts (venereal warts)							
Syphilis							
Trichomonas vaginalis (Trich, TV)							
Herpes (genital herpes)							
Pubic lice / crabs							
Hepatitis B							
(Men only:) NSU (Non Specific Urethritis), NGU (Non Gonococcal Urethritis)							
(Men only:) Epididymitis							
(Women only:) Pelvic Inflammatory Disease (PID, salpingitis)							
(Women							

only:) Vaginal thrush (Candida, Yeast infection)							
(Women only:) Bacterial vaginosis							
Yes, but can't remember which							
I was never tested of any. **(skip to HIV question D5)**							

<u>These are for dropdown menus</u>

***Where did you get the test from? 1. Public Hospital/clinic; 2. Private clinic/hospital; 3. CDC; 4. Online (buy testing kits online and test by yourself); 5. Pharmacy (buy testing kits at pharmacy and test by yourself); 6. Other (Specify _________)**

**** How did you know you had a STD? 1. Doctor's diagnosis; 2. Told by other people (not a doctor) ; 3. Self-testing/ diagnosis; 4. Had symptoms or pain; 5. Other (Specify _______)**

#When you had this last time, what did you do for treatment? 1. Went to a hospital ;

2. Visited a private clinic ; 3. Self-treatment ; 4. Took no medical measures

D2. When was the last time you had an STI??

1. During the past 12 months
2. More than one year ago but within four years
3. More than four years ago
4. Don't remember

D3. During the most recently time you had STD, how often did you use condoms?
1. Never
2. Rarely
3. Sometimes
4. Often
5. Always

D4. During the most recently time you had STD, how often did your partner use a condom?
1. Never
2. Rarely
3. Sometimes
4. Often
5. Always

D5. In China today, many people are infected with HIV, and they are infected with the diseases not just because of what they have done themselves. Up till now, have you ever been tested for HIV?
1. Yes
2. No **(skip to the next section)**
3. Don't know **(skip to the next section)**

D6. Where did you get the test from?

1. Public Hospital/clinic
2. Private clinic/hospital
3. CDC
4. Online (buy testing kits online and test by yourself)
5. Pharmacy (buy testing kits at pharmacy and test by yourself)
6. Other (Specify ______________________)

Stressful Events **(SE) (17 ITEMS)**

Listed below are a number of difficult or stressful things that sometimes happen to people. For each event check one or more of the boxes to the right to indicate whether: (a) it *happened to you* personally; (b) you *witnessed it* happen to someone else; (c) you *learned about it* happening to a close family member or close friend; (d) you were exposed to it as *part of your job* (for example, paramedic, police, military, or other first responder); (e) you're *not sure* if it fits; or (f) it *doesn't apply* to you.

Be sure to consider THE PAST ONE YEAR as you go through the list of events.

Event		*Happened to me*	*Witnessed it*	*Learned about it*	*Part of my job*	*Not Sure*	*Doesn't Apply*
1.	Natural disaster (for example, flood, hurricane, tornado, earthquake)						
2.	Fire or explosion						

3.	Transportation accident (for example, car accident, boat accident, train wreck, plane crash)						
4.	Serious accident at work, home, or during recreational activity						
5.	Exposure to toxic substance (for example, dangerous chemicals, radiation)						
6.	Physical assault (for example, being attacked, hit, slapped, kicked, beaten up)						
7.	Assault with a weapon (for example, being shot, stabbed, threatened with a knife, gun, bomb)						
8.	Sexual assault (rape, attempted rape, made to perform any type of sexual act through force or threat of harm)						
9.	Other unwanted or uncomfortable sexual experience						
10.	Combat or exposure to a war-zone (in the military or as a civilian)						
11.	Captivity (for example, being kidnapped, abducted, held hostage, prisoner of war)						
12.	Life-threatening illness or injury						
13.	Severe human suffering						

14.	Sudden violent death (for example, homicide, suicide)						
15.	Sudden accidental death						
16.	Serious injury, harm, or death you caused to someone else						
17.	Any other very stressful event or experience describe ______________						

SE18. Stressful events come in many forms. Among the ones you just listed, which was the most stressful and distressing experience you had?

____________(MOST STRESSFUL)

How much does reminders (thoughts, images, physical sensations) of this event interfere with:

		None	Mild	Moderate	Severe	Extreme or cannot do
**SE19a**	Maintaining social relationships?	1	2	3	4	5
**SE20b**	Your day-to-day work/school?	1	2	3	4	5

The interview is now complete. Thank you again for your support and help. Please return the computer to the interviewer. We wish you all the best!
