# Supplementary material for: Is there a relationship between geographic distance and uptake of HIV testing services? A representative population-based study of Chinese adults in Guangzhou, China
Source: PLoS One. 2017 Jul 20;12(7):e0180801. doi: 10.1371/journal.pone.0180801 (PMC5519047; doi:10.1371/journal.pone.0180801)
Supplement: S2 File — (ZIP) [file pone.0180801.s005.zip › questionnaire/Questionnaire-Chinese verision.pdf]

广州市居民健康调查问卷

入选标准:

您是在哪一年出生的?_ __________

(如果不是在1965-1996内出生，请排除)

参加者编号

__ __ __ __ __

进入调查的日期

__ __ / __ __ / __ __ __ __月/ 日/ 年

调查员编号

__ __ __

第一部分：背景及基本情况(B) (18 项)

B1. 您是？

1.男人　　　　　2.女人

B2. 您的民族?

1. 汉族　　　　　2. 其他少数名族（请说明__________. ）

B3. 您的文化程度是下列哪种？（各种学校都算；没毕业，也算是“上过”）

1. 没上过学　　2. 上过小学　　3. 上过初中

4. 上过高中、中专、职业高中　　5. 上过大专　　6.上过大学，或者研究生

99. 拒绝回答

B4. 您现在的婚姻状况是下列哪一种？

1. 没有结过婚，也没有试婚、同居　　2. 双方已经住在一起，还没有领结婚证

3. 已婚　　4. 已离婚　　5. 对方去世，没有再次结婚　　99.拒绝回答

B5. 您现在的住房状况是下列哪一种？

1.住自己买的房子　　2.自己租住一整套房子　　3.和别人一起合租

4.借住在别人家　　5.其他（请说明　　）　　99.拒绝回答

B6. 目前您和谁一起住？（多项选择）

1. 自己一个人住　　2. 和同事一起　　3. 和朋友一起住

4. 和配偶/恋人一起住　　5. 和父母一起住　　6.（只）和孩子一起住

7. 其他（请说明_____）　　99.拒绝回答

B7.大多数情况下，有多少人和您住在一起？ _______人

B8. 您住在您现在的街道（居委会）有多久了？

1. 0-3个月　2. 4-6个月　3. 7个月-1年　4. 1-5 年　5. >5年

B9. 您在广州生活了多长时间？

1. 0-3个月　2. 4-6个月　3. 7个月-1年　4. 1-5 年　5. >5年

B10. 您有广州户口吗？

1. 没有

2. 有

B11. 您的出生地（城市）_________________城市 __________县（如果是广州，请跳到 B13）

B12. 您搬到广州来的原因是

1. 工作原因　2. 和家人团聚　3. 学习　4. 其他原因 （请说明_____________）

B13. 过去5年，您在多少个地方生活过（包括城市，城镇，村庄）__________，时间超过三个月。

B14. 到广州之前，您上一个生活的地方是 __________

B15. 您目前的工作状况？您是

1. 有全职的工作　2. 有兼职的工作　3.目前没有工作，但正在找工作 （**跳至B17**）

4. 目前没有工作，也没有找工作(**跳至B17**)　5. 自己做生意　6. 其他（请说明）

77. 不知道　88. 不适用　99. 拒绝回答

B16. 现在，您主要是在做什么工作？

1. 农业生产者（农民）　2.工人，直接参加生产

3. 商业、服务业、娱乐业的普通工作者　4.个体劳动者、小商小贩等

5. 职员、一般干部、坐办公室的人、白领、警察

6. 科技人员、教师、知识分子、文艺界、传媒广告业

7. 各种企业家：经理、厂长等　8. 各级领导干部（包括村干部）

9. 以上都不是

B17. 在最近30天，您的个人总收入是______________元（尽量估计就好）

B18. 在最近30天，您的家庭总收入是______________ 元（尽量估计就好）

第二部分：一般健康状况 （G） (5项)

G1. 总的来说，您觉得自己的健康状况好不好？:

1. 非常好　　2. 比较好　　3. 一般，还可以　　4. 不太好　　5. 很不好

G2. 您的身高 ____ 厘米　　　　　G3. 您的体重 ________公斤

G4. 在过去的12个月中，医生或者其他专业人员有诊断过您患有什么疾病吗？

1. 有　　　　　2. 没有 （**若没有，跳转至第三部分**）

G5. 医生诊断您所患的疾病名称是： _____________ （可多选）

1. 后背疼痛持续三个月或三个月以上
2. 肌肉或骨骼方面疾病持续三个月或三个月以上
3. 除帕金森病和癫痫外，其他的精神病
4. 癌症　　　5. 甲状腺疾病　　　6. (卵巢/睾丸)或垂体的疾病
7. **仅女士回答**:我接受过试管受精或者其他生育治疗
8. 其他（ 请说明. _____________）

第三部分：健康行为 (HB) (29项)

HB1. 请回想过去的 30 天或以前，其中多少天你有吸烟？ 尽量估计 ____(范围：0 到 30，如果是 0，请跳至 HB3)

HB2.在您吸烟的日子中，您平均每天吸多少支烟尽量估计。____支/每天（一包= 20 支）

请阅读指导语:

下面一些问题是关于您最近 1 个月的睡眠情况，请选择或填写最符合您近 1 个月中大多数实际情况的答案。请回答下列问题！

HB3 近 1 个月，晚上上床睡觉通常是 ____点钟；

HB4 近 1 个月，从上床到入睡通常需要____分钟；

HB5 近 1 个月，通常早上____点起床；

HB6 近 1 个月，每夜通常实际睡眠_____小时（不等于卧床时间）；

HB7 近 1 个月，总的来说，您认为自己的睡眠质量

1. 很好　　2. 较好　　3. 较差　　4. 很差

HB8 请您说明睡眠问题对下列情况困扰的程度：

		从来不会	稍微有点	中等程度	情况严重	特别严重（几乎无法继续）
HB8a	保持良好的社会关系	1	2	3	4	5
HB8b	每天的工作或上学生活	1	2	3	4	5

请阅读指导语：

现在会问一些问题，关于过去的一年中，您饮用酒精饮料的情况。 一份酒精饮料==350ml（约等于 1 瓶）的啤酒==150ml(半高脚杯)葡萄酒 ==50ml（约等于 1 两）的烈酒或白酒

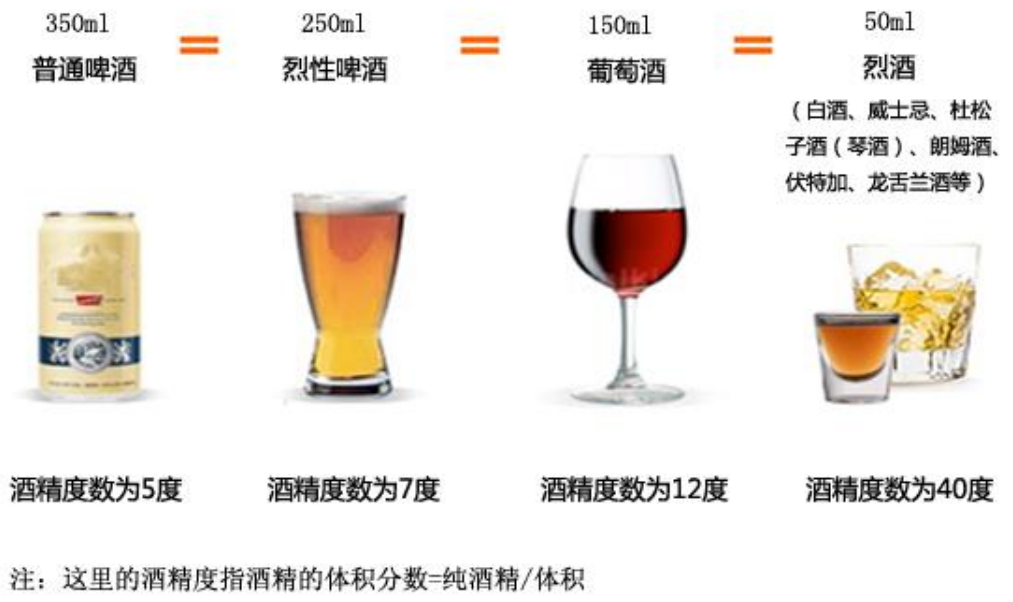

HB9.您喝酒的次数是多少？（单选）

0.从不（**跳到HB19**）　1.每月约一次　2.每月2—4次　3.每周2—3次

4.每周4次以上　77.不知道　88.不适用　99.拒绝回答

HB10.在喝酒的那一天中所饮的酒量为多少“杯”？（单选）

1.　1或2杯　2.　3或4杯　3.　5或6杯　4.　7或9杯

5.　10杯以上　77.不知道　88.不适用　99.拒绝回答

HB11.每次喝6杯以上的次数为多少？（单选）

0.从不　1.每月不到一次　2.每月一次　3.几乎每周一次

4.每天或者几乎每天　77.不知道　88.不适用　99.拒绝回答

HB12. 你是否一开始喝酒就无法立即中断？这种情况在最近一年中有几次？

0.从不　　　1.每月不到一次　　　2.每月一次　　　3.几乎每周一次

4.每天或者几乎每天　　　77.不知道　　　88.不适用　　　99.拒绝回答

HB13. 你有没有因为喝酒而贻误了该做的事情？这种情况在最近一年中有几次？

0.从不

1.每月不到一次

2.每月一次

3.几乎每周一次

4.每天或者几乎每天

77.不知道

88.不适用

99.拒绝回答

HB14. 在一次大量饮酒后，你是否需要再次日早上喝一些酒才能正常生活？这种情况在最近一年中有几次？

0.从不

1.每月不到一次

2.每月一次

3.几乎每周一次

4.每天或者几乎每天

77.不知道

88.不适用

99.拒绝回答

HB15.你会不会在饮酒之后感到内疚或者后悔？这种情况在最近一年中有几次？

0.从不

1.每月不到一次

2.每月一次

3.几乎每周一次

4.每天或者几乎每天

77.不知道

88.不适用

99.拒绝回答

HB16. 你会不会因为喝酒而回忆不起前夜所发生的情况？这种情况在最近一年中有几次？

0.从不

1.每月不到一次

2.每月一次

3.几乎每周一次

4.每天或者几乎每天

77.不知道

88.不适用

99.拒绝回答

HB17.有没有因为你喝酒而使你本人或者他人受到损伤的情况？

0.没有

1.有，但是不在过去的一年

2.有，是在过去的一年

77.不知道

88.不适用

99.拒绝回答

HB18.你的亲戚好友、医生或者其他卫生工作者有没有关心过你的饮酒问题，并劝过你戒酒？

0.没有

1.有，但是不在过去的一年

2.有，是在过去的一年

77.不知道

88.不适用

99.拒绝回答

请阅读指导语：

现在我们想了解一些关于您药物使用方面的情况。

HB19. 请您说明，在上个月，您是否使用过下列药物；或者曾经使用过下列药物。如果使用过，请问您你否在发生性行为之前或者在发生性行为的过程中使用过下列药物。

药物	上个月使用过么？	上个月，在发生性行为之前或者在发生性行为的过程中使用过么？	如果在上个月没有使用过：您曾经使用过么？	如果曾经使用过：在发生性行为之前或者在发生性行为的过程中使用过么？
1. 海洛因	0	1	2	
2. 古柯碱	0	1	2	
3. 甲基安非他命	0	1	2	
4. 恺他命	0	1	2	
5. 吗啡	0	1	2	
6. 大麻	0	1	2	
7. 哌替啶盐酸盐	0	1	2	
8. 丁基原啡因舌下锭	0	1	2	
9. 苯环利定	0	1	2	
10. 酒精	0	1	2	
11. 其他，请说明 ____________	0	1	2	

科学名称	俗称
**海洛因**	白粉、白面儿、四号、四号仔、软仔、小仔
**古柯碱**	可卡因、可可、快克、crack、snow
**安非他命/［甲基苯丙胺/甲基安非他命］**	猪肉、安公子、安仔、冰糖、冰块、盐、Speed、糖果、冰毒
**恺他命**	卡门、K、K他命、Special K、K粉、克他命、K仔
**吗啡**	魔啡
**大麻**	草、饭、麻仔、老鼠尾
**哌替啶盐酸盐**	杜冷丁、度冷丁、哌啶、配西汀、地美露（Demerol）、盐酸哌替啶
**丁基原啡因舌下锭**	布诺啡、叔丁啡、丁苯诺啡、Temegesic、Buprenex
**苯环利定**（PCP）	天使尘、Love boat
**摇头丸**	忘我、快乐丸、绿蝴蝶、亚当、狂喜、衣服、Eve、夏娃

第四部分：心理健康(MH)（24项）

MH1. 医生或者其他专业人员有诊断过您患有什么心理方面的疾病吗？

1. 有

0. 没有 （**选此答案，请跳到第五部分**）

77.不清楚

88. 拒绝回答

MH2. 医生诊断您所患心理方面的疾病名称是：______________________________

MH3. 在过去的 3 个月中，您有过下列症状么？

**症状**	**没有**	**有**
1.用脑后倍感疲倦的持续而痛苦地主诉（向别人说）	0	1
2.轻度用力后身体虚弱与极度疲倦的持续而痛苦地主诉（向别人说）	0	1
3. 肌肉疼痛感，头昏	0	1
4.紧张性头痛	0	1
5.睡眠紊乱	0	1
6.不能放松，易激惹	0	1
7.消化不良	0	1

MH4.上述问题有多大程度会困扰到您:

		从来没有	轻微影响	中等影响	严重影响	十分严重或者无法解决
MH4a	维持社会关系	1	2	3	4	5
MH4b	每天的工作和学习生活	1	2	3	4	5

MH5. PHQ-9 (抑郁量表):

请回忆过去 2 周的时间中，您有多少时间出现过下述情况：

问题	完全不会	好几天	一半以上的天数	几乎每天
1、做事时提不起劲或没有兴趣	0	1	2	3
2、感到心情低落、沮丧或绝望	0	1	2	3
3、入睡困难、睡不安或睡眠太多	0	1	2	3
4、感觉疲倦或没有活力	0	1	2	3
5、食欲不振或吃太多	0	1	2	3

6、觉得自己很糟—或觉得自己很失败，或让自己或家人失望	0	1	2	3
7、对事物专注有困难，例如阅读报纸或看电视时	0	1	2	3
8、动作或说话速度缓慢到别人已经觉察。或正好相反—烦躁或坐立不安、动来动去的情况更胜于平常	0	1	2	3
9、有不如死掉或用某种方式伤害自己的念头	0	1	2	3

MH6.上述问题有多大程度会困扰到您:

		从来没有	轻微影响	中等影响	严重影响	十分严重或者无法解决
MH6a	维持社会关系	1	2	3	4	5
MH6b	每天的工作和学习生活	1	2	3	4	5

MH7.过去的12个月，你曾经向下面所列人寻求过帮助去解决心理或精神的问题:(可以多选)

1. 没有寻求过帮助 （**请跳到第五部分.**）
2. 精神病医生
3. 心理咨询师
4. 其他医生
5. 社会工作者
6. 其他人,（请说明 ______________）

MH8.请描述您寻求这个人（ITEM MH7选择的人）的原因. ______________

第五部分：社会支持与信任 (SC) （14项）

SC1. 您有多少关系密切，可以从他们那儿得到支持和帮助的朋友？

① 一个也没有 ② 1-2 个 ③ 3-5 个 ④ 6 个及以上

SC2. 近一年来您：

① 远离家人，且独居一室 ② 住处经常变动，多数时间和陌生人在一起

③ 和同学、同事或朋友在一起　④ 和家人在一起

SC3. **您与邻居：**

① 相互之间从不关心，只是认识　② 遇到困难可能稍微关心

③ 有些邻居都很关心您　④ 大多数邻居都很关心您

SC4. **您与同事：**

① 相互之间从不关心，只是认识　② 遇到困难可能稍微关心

③ 有些同事都很关心您　④ 大多数同事都很关心您

SC5. **从家庭成员处得到的支持和照顾（在合适的框内划“√”）**

	**有/无该家庭成员**	**从该家庭成员处得到的支持和照顾**			
		**无**	**极少**	**一般**	**全力支持**
		0	1	2	3
夫妻（恋人）	有/无				
父母	有/无				
儿女	有/无				
兄弟姐妹	有/无				
其他成员（如嫂子）	有/无				

SC6. **在您遇到急难情况时，曾经得到的经济支持和能够解决实际问题的帮助来源有：**

① 无任何来源

② 下列来源：**（可多选）**

1） 配偶　2） 其他家人（不包括配偶）　3）朋友　4）亲戚　5）同事

6）工作单位 7） 党团工会等官方或半官方组织 8）宗教、社会团体等非官方组织

9）其他（**请列出：______________________________**）

SC7.　**在您遇到急难情况时，曾经得到的安慰和关心的来源有：**

①　无任何来源

②　下列来源：**（可多选）**

1）　配偶　　2）其他家人（不包括配偶）　3）朋友　4）亲戚　　5）同事

6）工作单位　7）　党团工会等官方或半官方组织　8）　宗教、社会团体等非官方组织

9）其他（**请列出：____________________________**）

SC8.　您遇到烦恼时的倾诉方式：

① 从不向任何人倾诉　　　　　　② 只向关系极为密切的 1-2 个人倾诉

③ 如果朋友主动询问，您会说出来　④ 主动倾诉自己的烦恼，以获得支持和理解

SC9.　您遇到烦恼时的求助方式：

① 只靠自己，不接受别人帮助　　　② 很少请求别人帮助

③ 有时请求别人帮助　　　　　　④ 有困难时经常向家人、亲友、组织求援

SC10. 对于团体（如党团组织、宗教组织、工会、学生会等）组织活动，您：

① 从不参加　　② 偶尔参加　　③ 经常参加　　④ 主动参加并积极活动

现在我们想了解一下您对所在地的人群的看法

SC11.　"在这个村或者地区大部分的人是诚实并且能被信任的。"您同意这个观点么？

1. 非常不同意

2. 不同意

3. 同意

4. 非常同意

以下询问流动人口（没有广州户口的人）

SC12.　"我觉得广州本地人能够接纳我'你同意这个观点么？"

1. 非常不同意

2. 不同意

3. 同意

4. 非常同意

以下询问非流动人口（有广州户口的人）

SC13.“我认为来自其他省的流动人口，不应该住在广州。” 你同意这个观点么？

1. 非常不同意

2. 不同意

3. 同意

4. 非常同意

SC14.“我认为来自其他省的流动人口，不应该获得本地的户口”你同意这个观

1. 非常不同意

2. 不同意’

3. 同意

4. 非常同意

现在我们想了解一下您的性行为和性健康

第六部分：性伴侣(S)（10项）

S

S1. 请您说明您的性取向：

1. 异性恋

2. 同性恋

3. 双性恋

4. 不确定

S

S2. 到现在为止，你曾经与其他人有过性生活吗（通过阴道）(包括仅与某人发生过一次的情况)

1. 有过，经常

2. 有过，有些时候

3. 有过，但是很少

4. 从来没有

S3.如今，许多人即使与异性也发生肛交。您有没有觉得这些事很脏？？

1. 有过

2. 没有

SS4. 您曾经有没有与性伴侣发生过肛交？（只有一次也算)？

1. 有过，经常

2. 有过，有些时候

3. 有过，但是很少

4. 从来没有(请跳到**第九部分：性传播疾病**)

S5. 目前（过去一年内），您与别人有性关系吗？【提示语：包括通过阴道和肛门，和任何人，都请包括进来】

1. 有
2. 没有（**选此答案，接下来回答第九部分：性传播疾病**）

S6. 目前（过去一年内），您与多少人发生过性关系？（提示语：包括通过阴道和肛门）

1. 1 个

2. 2~3 个（**跳至 S8**）

3. 4~5 个(**跳至 S8**)

4. >5 个（**跳至 S8**）

S7. 现在与您有性关系的这个人，与您住在一起吗？

1. 是，住在一起

2. 不是（**选此答案，跳至第八部分:短期性伴侣**）

S7a.这个人是男人还是女人？

1. 男人(**跳至 C1, 然后做 C2-1**)

2. 女人（**跳至C1, 然后做 C2-2**)

S8. 您刚才说，现在跟您保持着性关系的人，有一个以上。那么，其中有与您住在一起的吗？

1. 是，住在一起

2. 不是（**选此答案，跳至第八部分:短期性伴侣**）

S8a. 这个人是男人还是女人？

1. 男人(**跳至 C1, 然后做 C2-1**)

2. 女人（**跳至C1, 然后做 C2-2**)

第七部分：目前的性伴侣（C)（9 项）

C1. **我们想了解一下目前与您有性关系而且住在一起的人，无论是丈夫、妻子、同居的人，还是其他性关系都算**

C2-1. 现在与您有性关系的这个男人，请您为他选择一个最恰当的称呼？【提示语：只适用于 S6=1 的人回答】

1. 我的爱人，老公

2. 我的男友

3. 我的情人

4. 熟人、朋友

5. 一般朋友

6. 网友

7. 我的老板

8. 上级

9. 工作业务来往中的客户 s

10. 顾客（特指购买性服务的客人）

11. 以上都不是，是其他情况

C2-2. 现在与您有性关系的这个女人，请您为他选择一个最恰当的称呼？【提示语：只适用于 S6=2 的人回答】

1. 我的爱人、老婆

2. 我的女友

3. 二奶

4. 熟人、朋友

5. 一般朋友

6. 网友

7. 小姐（特指提供性服务的人）

8. 以上都不是，是其他情况

C3. 您与这个人的性关系，已经保持了多长时间？

1.不到一个月

2. 1~ 6 个月

3. 7~12 个月

2. 不到两年

3. 2 ~ 4 年

4. 超过 4 年

C4. 您和他/她保持性关系的期间，您自己还跟别的人有过性生活吗？（提示语：哪怕只有一次，也算有过。）

1. 有过

2. 没有

C5. 您的这个性伴侣，在你们保持性关系期间，是不是跟别的人有过性生活？【提示语：哪怕只有一次，也算有过。】

1. 是的，肯定有

2. 也许有，我无法知道

3. 肯定没有

C6. 您与这个性伴侣，上一次过性生活，是在多长时间以前？

1.一个星期内

2.两个星期内

3.一个月内

4.超过一个月，但是在一年内

5.超过一年［**请跳至第九部分：性传播疾病**］

C7. 在过去的 12 个月内，在您们过性生活的期间，平均多长时间过一次性生活？

1. 每天一次或以上

2. 每周三至六次

3. 每周一至两次

4. 每月两至三次

5. 每月一次或以下

C8. 您与这个性伴侣经常使用避孕套吗？

1. 总是用 （请跳至第九部分：性传播疾病）

2. 经常用

3. 很少用

4. 从来没用过（请跳至第九部分：性传播疾病）

C9. 你们上一次过性生活时，使用避孕套了吗？

1. 用了

2. 没用

第八部分:短期性伴侣（ST）（12 项）

ST1. 在过去的 4 个星期里，总共有多少人和您发生了性行为？_________人

【提示语：无论什么人都包括，也包括长期性伴侣。如果一个都没有，回答 0，然后回答第九部分】

ST2. 在过去的四个星期里，您有多少个新的性伴侣？_________人

【提示语：也就是说，您是第一次和她（他）发生性行为的，总共有多少个？，无论什么人都包括。如果一个都没有，请回答：0】

ST3. 下面请您说说：除了与您住在一起的爱人之外，最近跟您有性生活的那个人。

请问：这个人是男人，还是女人？

1. 男人（**选此答案，接下来回答 ST4 题**）
2. 女人（**选此答案，接下来回答 ST5 题**）

ST4. 现在与您有性关系的这个男人，请您为他选择一个最恰当的称呼？【提示语：只适用于 ST3=1 的人回答】

1. 男朋友
2. 未婚夫
3. 前夫
4. 情人
5. 熟人、朋友
6. 一般朋友
7. 网友
8. 老板
9. 上级
10. 工作业务来往中的客户
11. 顾客（特指购买性服务的客人）
12. 性伴、性伙伴、性伴侣
13. 以上都不是，是其他情况

ST5. 现在与您有性关系的这个女人，请您为他选择一个最恰当的称呼？【提示语：只适用于 ST3=2 的人回答】

1. 女朋友
2. 未婚妻
3. 二奶
4. 熟人、朋友
5. 一般朋友
6. 网友
7. 小姐（特指提供性服务的人）
8. 前妻
9. 性伴、性伙伴、性伴侣
10. 以上都不是，是其他情况

ST6. 到目前为止，您与他/她总共发生过多少次性行为？__________次【提示语：回答≥2，接下来回答 ST8 题】

ST7. 您与他/她发生性行为时，使用避孕套了吗？【提示语：只有一次也算，且本题只适合上一题回答“1”的人回答。答完本题，接下来回答 ST9 题】

1. 用了
2. 没用

ST8. 您与这个性伴侣经常使用避孕套吗?

1. 总是用
2. 经常用
3. 很少用
4. 从来没用过

ST9. 您与这个性伴侣，上一次过性生活，是在多长时间以前？

1. 一个星期以内

2. 两个星期以内

3. 四个星期以内

4. 四个星期以前(请参加者再次回忆,因为本部分是调查四个星期内最长发生性关系的性伴侣)

ST10. 您与这个人的性关系，保持了多长时间？

1. 不到一周

2. 不到一个月

3. 1—3 个月

4. 4—6 个月

5. 7—12 个月

6. 超过一年

7. 超过四年

ST11. 您和他/她保持性关系的期间，您自己还跟别的人有过性生活吗？【提示语：哪怕只有一次，也算有过。】

1. 有过

2. 没有

ST12. 您的这个性伴侣，在你们保持性关系期间，是不是跟别的人有过性生活？【提示语：哪怕只有一次。】

1. 是的，肯定有

2. 也许有，我无法知道

3. 肯定没有

第九部分：性传播疾病 (D)（24 项）

D0. 在您的一生里，您一共跟多少个人有过性生活？________人.【提示语：哪怕只有一次也算，无论什么人都算，包括结婚的、没结婚的、离婚或分手的；也包括临时的、偶然的（例如“小姐”）；还包括同性。】

D1. 目前在中国，得过性病的人已经很多了，他们得病不只是因为他们自己的行为。请问，到现在为止，您曾经接受过性病检测吗？

STDs	**D1a.** 从来没有做过检测(跳至下一行)	**D1b.** 有过	**D1c*.** 请问您是在哪里做的检查?	**D1d.** 在您的一生里，您是否得过这个疾病(如果没有请选 2，并跳至下一题)	**D1e**.** 您如何知道您得了这个疾病**?**	**D1f.** 在过去的12个月中，我被告知得这个病的次数是？	**D1g#.** 最后一次您得这个疾病的时间是？您采取了什么治疗方式？
衣原体							
淋病							
生殖器疣(性病湿疣)							
梅毒							
阴道毛滴虫							
生殖器疱疹							
阴虱							
乙型肝炎							
**仅针对男性:** (非特异性尿道炎，非淋球菌尿道炎)							
**仅针对男性:** 附睾炎							
**仅针对女性:** 盆腔炎(输卵管炎)							
**仅针对女性:**阴道真菌感染(念珠菌,酵母菌感染)							
**仅针对女性:** 细							

菌性阴道病							
有过，但是不记得是哪一种了							
我从来没有检测过(**请跳至 HIV的问题 D5**)							

***您在哪里做的检测？**1. 公立医院/诊所; 2.私人医院/诊所; 3. CDC（疾病控制中心）; 4. 在网上购买检测工具，然后自己检测; 5. 在药店购买检测用品，自己检测); 6. 其他（请说明）

**** 您如何知道您得了性传播疾病** 1.医生的诊断 2. 其他人告诉我的 (不是医生) ; 3. 自己检测/诊断; 4. 有相关症状和疼痛 5. 其他 (请说明 ________)

#最后一次您得这个疾病的时间是？您采取了什么治疗方式？1.去医院 ; 2. 去私人诊所; 3. 自己治疗 ; 4. 没有采取治疗

D2. 您最近一次得性病，是多久以前的事？

1. 过去 12 个月内
2. 1 年以上，4 年以内
3. 4 年以前
4. 记不清

D3. 在您最近一次得性病的那段时间之内，您自己使用安全套（避孕套）了吗？

1. 不，一直没有使用过
2. 很少用
3. 有时用，有时不用
4. 用得比较多
5. 很经常使用

D4. 在您最近一次得性病的那段时间之内，他(对方)使用安全套（避孕套）了吗？

1. 不，一直没有使用过

2. 很少用

3. 有时用，有时不用

4. 用得比较多

5. 很经常使用

D5. 目前在中国，得过 HIV 的人已经很多了，他们得病不只是因为他们自己的行为。请问，到现在为止，您曾经接受过 HIV 检测吗？

1. 有过

2. 没有(**请跳至第十部分：压力事件**)

3. 不知道(**请跳至第十部分：压力事件**)

D6. 您在哪里做的检测？

1. 公立医院/诊所;

2. 私人医院/诊所;

3. CDC（疾病控制中心）;

4. 在网上购买检测工具，然后自己检测;

5. 在药店购买检测用品，自己检测);

6. 其他（请说明____________________）

应激事件(SE) (17 项)

指导语：下边列出了一些人们可能遇到的应激性事件。每个事件都有以下五个选项：1. 发生在您身上；2. 您目击了该事件；3. 您知道该事件发生在与您关系亲密的人身上；4. 您不确定该事件是否适用于您；5.该事件不适用于您。请您仔细阅读每一个事件，选择一个最适用于您的选项，并在该选项上画“○”。

另外，请确定当您回答问题时，思考的是您**过去一年中的事情**。

	经历过	目击过	知道	不确定	不适用
1. 自然灾害（如洪水、飓风、龙卷风、地震）	1	2	3	4	5

2. 火灾/爆炸	1	2	3	4	5
3. 交通事故（如车祸、航海事故、火车事故、飞机失事）	1	2	3	4	5
4. 在工作、住宅或者是休闲运动中遇到严重的事故	1	2	3	4	5
5. 暴露于有毒物质（如危险化学物质、放射性物质）	1	2	3	4	5
6. 人身攻击（如被攻击、撞击、扇耳光、踢、痛打）	1	2	3	4	5
7. 使用武器袭击（如被枪击、刺伤或使用刀、枪、炸弹威胁）	1	2	3	4	5
8. 性侵犯（强奸、强奸未遂、被暴力威胁或威胁伤害导致任何类型的性行为）	1	2	3	4	5
9. 其他不想要的或非意愿的性经历	1	2	3	4	5
10. 经历战争或曾经在战争地区（作为平民或者是军人）	1	2	3	4	5
11. 被囚禁（如被诱拐、绑架、成为人质、战争的囚犯）	1	2	3	4	5
12. 威胁生命的疾病或外/内伤	1	2	3	4	5
13. 严重的苦难	1	2	3	4	5
14. 突然死亡，横死（如杀人、自杀）	1	2	3	4	5
15. 亲密的人突然的、意料之外的死亡	1	2	3	4	5
16. 因为您而导致其他人严重受伤、伤害或者是死亡	1	2	3	4	5
17. 其他的应激性事件或经历	1	2	3	4	5

SE18.生活中的应激事件有不同的形式. 在您所选择的应激事件中,哪一种是您所经历过压力最大，并且是最痛苦的呢？

____________(压力最大)

请您说明上列记忆（回想，画面，身体感觉）困扰您的程度：

		从来不会	稍微有点	中等程度	情况严重	特别严重（几乎无法继续）
**SE19a**	保持良好的社会关系	1	2	3	4	5
**SE20b**	每天的工作或上学生活	1	2	3	4	5

调查到此结束，再次感谢您的参与和配合！请您将调查仪器交还给调查员！祝您身体健康，万事如意！
